# Comparative Genomics Underlines Multiple Roles of *Profftella*, an Obligate Symbiont of Psyllids: Providing Toxins, Vitamins, and Carotenoids

**DOI:** 10.1093/gbe/evaa175

**Published:** 2020-08-14

**Authors:** Atsushi Nakabachi, Jörn Piel, Igor Malenovský, Yuu Hirose

**Affiliations:** e1 Electronics-Inspired Interdisciplinary Research Institute (EIIRIS), Toyohashi University of Technology, Japan; e2 Department of Applied Chemistry and Life Sciences, Toyohashi University of Technology, Japan; e3 Institute of Microbiology, Eidgenössische Technische Hochschule (ETH) Zürich, Zurich, Switzerland; e4 Department of Botany and Zoology, Faculty of Science, Masaryk University, Brno, Czechia

**Keywords:** *Diaphorina*, defensive symbiont, reduced genome, secondary metabolite, diaphorin, hemolysin

## Abstract

The Asian citrus psyllid *Diaphorina citri* (Insecta: Hemiptera: Psylloidea), a serious pest of citrus species worldwide, harbors vertically transmitted intracellular mutualists, *Candidatus* Profftella armatura (*Profftella*_DC, Gammaproteobacteria: Burkholderiales) and *Candidatus* Carsonella ruddii (*Carsonella*_DC, Gammaproteobacteria: Oceanospirillales). Whereas *Carsonella*_DC is a typical nutritional symbiont, *Profftella*_DC is a unique defensive symbiont with organelle-like features, including intracellular localization within the host, perfect infection in host populations, vertical transmission over evolutionary time, and drastic genome reduction down to much less than 1 Mb. Large parts of the 460-kb genome of *Profftella*_DC are devoted to genes for synthesizing a polyketide toxin; diaphorin. To better understand the evolution of this unusual symbiont, the present study analyzed the genome of *Profftella*_Dco, a sister lineage to *Profftella*_DC, using *Diaphorina* cf. *continua*, a host psyllid congeneric with *D. citri*. The genome of coresiding *Carsonella* (*Carsonella*_Dco) was also analyzed. The analysis revealed nearly perfect synteny conservation in these genomes with their counterparts from *D. citri*. The substitution rate analysis further demonstrated genomic stability of *Profftella* which is comparable to that of *Carsonella*. *Profftella*_Dco and *Profftella*_DC shared all genes for the biosynthesis of diaphorin, hemolysin, riboflavin, biotin, and carotenoids, underlining multiple roles of *Profftella*, which may contribute to stabilizing symbiotic relationships with the host. However, acyl carrier proteins were extensively amplified in polyketide synthases DipP and DipT for diaphorin synthesis in *Profftella*_Dco. This level of acyl carrier protein augmentation, unprecedented in modular polyketide synthases of any known organism, is not thought to influence the polyketide structure but may improve the synthesis efficiency.

**Significance**

*Profftella* is a highly unique bacterial symbiont found in the Asian citrus psyllid, a notorious agricultural pest. It has features like organelles but unusually produces toxins to protect the host insect. To better understand the evolution of this enigmatic symbiont, we sequenced the genome of a novel strain of *Profftella* recently found in another psyllid species. The comparative analysis of the two *Profftella* strains revealed that they share genes for the biosynthesis of toxins, vitamins, and carotenoids, highlighting multiple roles of *Profftella*. These collective benefits to the host may contribute to the stabilization of symbiotic relationships, leading to the organelle-like status of *Profftella*. The analysis also revealed unusual structures of toxin-producing enzymes, which may potentially improve the toxin production efficiency.

## Introduction

Animals exhibit diverse symbiotic relationships with microbes, among which the most intimate ones predominantly have a nutritional basis (Moran et al. 2008; McFall-Ngai et al. 2013; Morris et al. 2019). For animals feeding only on diets that are deficient in essential nutrients, supplementation of nutritional deficiencies is essential for their survival. Thus, microbes that are able to supply such nutrients can easily become vital to the host animals, resulting in evolutionarily stable nutritional symbionts. Extreme cases are exemplified by bacteriome-associated symbionts in insects (Douglas 1989; Moran et al. 2008; Moran and Bennett 2014; McCutcheon et al. 2019). Various insect lineages that feed on nutritionally restricted diets, including plant sap and vertebrate blood, have a specialized organ called a bacteriome, and depend on nutritional supply by “primary symbionts” housed therein. The primary symbionts are taxonomically diverse in distinct host lineages (e.g., aphids, weevils, tsetse flies, etc.), indicating that they have evolved repeatedly from diverse free-living microbes (Moran et al. 2008; Moran and Bennett 2014; McCutcheon et al. 2019). They are mostly bacterial and are characterized by organelle-like features, including intracellular localization within the host cell, perfect infection in host populations, host–symbiont cospeciation reflecting strictly vertical transmission over evolutionary time, and drastic genome reduction down to <1 Mb (Moran et al. 2008; Moran and Bennett 2014; McCutcheon et al. 2019). Mutually indispensable associations between the hosts and the primary symbionts have been demonstrated by physiological (Nogge 1981; Douglas 1989; Nakabachi and Ishikawa 1999) and omics analyses (Nakabachi et al. 2005; Moran et al. 2008; Ramsey et al. 2010; Shigenobu et al. 2010; Moran and Bennett 2014; Wilson and Duncan 2015; McCutcheon et al. 2019). In some cases, metabolic complementarity is achieved, at least in part, through horizontal gene transfer between the host and symbionts (Nikoh and Nakabachi 2009; Nikoh et al. 2010; Husnik et al. 2013; Sloan et al. 2014; Nakabachi et al. 2014; Nakabachi 2015; Mao et al. 2018).

Another major category of animal-associated microbes is represented by defensive symbionts (Piel 2009; Flórez et al. 2015; Helfrich and Piel 2016; Rust et al. 2020). They protect hosts from natural enemies, including predators, parasites, parasitoids, and microbial pathogens, using biologically active compounds such as toxins and antibiotics. In contrast to nutritional symbionts, the status of defensive symbionts tends to be unstable, probably because 1) they are only conditionally beneficial and not essential to the host, 2) the status of natural enemies in the environment can vary continuously, and 3) defensive toxins potentially place a burden on the host. Thus, most defensive symbionts, reported from various insects and marine invertebrates thus far, exhibit imperfect infection frequencies in host populations (Moran et al. 2008; Piel 2009; Oliver et al. 2010; Kwan et al. 2012; Flórez et al. 2015; Johnson 2015; Ballinger and Perlman 2019), and their genomes are often larger than 1 Mb (Wu et al. 2004; Degnan et al. 2009; Kwan et al. 2012; Wilson et al. 2014; Lopera et al. 2017; Flórez et al. 2018; Waterworth et al. 2020), even when they can be vertically transmitted. By contrast, an unprecedented type of defensive symbiont was found in the Asian citrus psyllid, *Diaphorina citri* (Hemiptera: Psylloidea: Liviidae).

*Diaphorina citri* is an important agricultural pest that transmits *Candidatus* Liberibacter spp. (Alphaproteobacteria: Rhizobiales), which cause a devastating disease of citrus crops known as huanglongbing or greening disease (Grafton-Cardwell et al. 2013). *Diaphorina citri* possesses a bacteriome (Nakabachi et al. 2010) that harbors two distinct species of vertically transmitted intracellular symbionts: *Candidatus* Carsonella ruddii (Gammaproteobacteria: Oceanospirillales) and *Candidatus* Profftella armatura (Gammaproteobacteria: Burkholderiales) (Nakabachi, Ueoka, et al. 2013; Dan et al. 2017). The primary symbiont *Carsonella*, which is thought to be present in all psyllid species, is a typical nutritional symbiont, providing its host with essential amino acids that are scarce in the diet of phloem sap (Nakabachi et al. 2006; Sloan and Moran 2012b; Nakabachi, Ueoka, et al. 2013). The secondary symbiont *Profftella* is found in all *D. citri* individuals across the world and has a very much reduced genome of 460 kb (Nakabachi, Ueoka, et al. 2013). Although this is generally characteristic of nutritional symbionts associated with bacteriomes (Moran et al. 2008; Moran and Bennett 2014; McCutcheon et al. 2019), the genome of *Profftella* encodes only a few genes required to complement the psyllid diet. Instead, large parts of the genome are composed of genes for synthesizing a secondary metabolite; diaphorin (Nakabachi, Ueoka, et al. 2013). Diaphorin is a polyketide belonging to the pederin family of cytotoxins that are found in a diverse array of host–symbiont systems, including beetles, lichens, and sponges harboring phylogenetically diverse bacterial producers (Helfrich and Piel 2016; Rust et al. 2020). Physiological studies have shown that diaphorin is significantly toxic to various organisms, including natural enemies of *D. citri* (Nakabachi, Ueoka, et al. 2013; Nakabachi and Fujikami 2019; Nakabachi and Okamura 2019; Yamada et al. 2019). Therefore, *Profftella* is considered to be an unprecedented type of defensive symbiont with organelle-like features. Moreover, the *Liberibacter* lineage, except for the most basal species *Liberibacter crescens*, is shown to have horizontally acquired a gene for a putative amino acid transporter from the *Profftella* lineage (Nakabachi, Nikoh, et al. 2013). This indicates ecological and evolutionary interactions between the huanglongbing pathogen and the bacteriome symbiont. Thus, comparative genomics of *Profftella* lineages aiming to better understand the evolutionary trajectory of this unique symbiont is desired.

Our previous study using Illumina sequencing of 16S rRNA gene amplicons demonstrated that *Diaphorina* cf. *continua*, a psyllid species closely related to *D. citri*, possesses a bacterial lineage that is sister to the *Profftella* of *D. citri* (Nakabachi et al. 2020). *Diaphorina citri* and *D*. cf. *continua* are different in their geographical distributions and host plants. Whereas *D. citri* is native to tropical and subtropical South to East Asia, and absent from Europe (Grafton-Cardwell et al. 2013), *D*. cf. *continua* occurs in the Mediterranean region (Nakabachi et al. 2020). Also, whereas *D. citri* feeds and develops on rutaceous plants including *Citrus* spp. (Grafton-Cardwell et al. 2013), *D*. cf. *continua* is associated with *Thymelaea tartonraira* (Thymelaeaceae), which is distantly related to the Rutaceae (Nakabachi et al. 2020). In the present study, to obtain insights into the evolution of *Profftella*, the genome of *Profftella* from *D.* cf. *continua* (*Profftella*_Dco) was analyzed along with that of *Carsonella* (*Carsonella*_Dco).

## Materials and Methods

### Insects and DNA Preparation

The material of *Diaphorina* cf. *continua* was collected in Corsica island (France, Haute-Corse department) near Moltifao village (42°29′12″N, 9°8′22″E, 300 m a.s.l.) on April 9, 2017. Many adults and several nymphs were present on *T. tartonraira* subsp*. thomasii* (Thymelaeaceae), suggesting that this plant taxon is the host for the psyllid species. The specimens have been tentatively identified as *D. continua* Loginova, based on morphology and host plant data (Loginova 1972; Burckhardt 1984; Rapisarda 1991; Conci et al. 1993), but their identity needs to be confirmed by a taxonomic revision (Nakabachi et al. 2020). DNA was extracted from whole bodies of mixed sex (three males and eight females) of adult *D.* cf. *continua* using a DNeasy Blood & Tissue Kit (Qiagen, Hilden, Germany) following the manufacturer’s instructions. The quality of extracted DNA was assessed using a NanoDrop 2000c spectrophotometer (Thermo Fisher Scientific, Waltham, MA), and the quantity was assessed using a Qubit 2.0 Fluorometer with a Qubit dsDNA HS Assay Kit (Thermo Fisher Scientific).

### Sequencing and Assembly

DNA extracted from *D.* cf. *continua* was sheared to ∼500 bp using a Covaris M220 Focused-ultrasonicator (Covaris, Woburn, MA). After a paired-end library was generated with 15 cycles of polymerase chain reaction amplification using a KAPA HyperPrep Kit (KAPA Biosystems, Wilmington, MA), 300 bp of each end of the library was sequenced using a MiSeq instrument (Illumina, San Diego, CA) and MiSeq Reagent Kit v3 (600 cycles; Illumina). Subsequently, BlastN searches were performed using the obtained reads as queries and the genome sequences of *Carsonella* from ten psyllid lineages (AP009180.1, CP003541.1, CP003542.1, CP003543.1, CP003544.1, CP003545.1, CP003467.1, CP012411.1, CP019943.1, CP024798.1) and *Profftella* from two strains of *D. citri* (chromosome: CP003468.1 and CP012591.1; plasmid: CP003469.1 and CP012592.1) as subjects. Read pairs with *e*-value scores lower than 1.0E-5 in either read, and those with GC content below 30% were collected. Adapters of filtered reads were removed using cutadapt (Martin 2011) with -m 10 -e 0.2 options. After sequencing errors were corrected using bfc (Li 2015) with default parameters, the reads were assembled using Newbler v2.9 (Roche Diagnostics, Rotkreuz, Switzerland) with -mi 99 -ml 100 -large options. Obtained scaffolds and contigs were manually combined using GenoFinisher (Ohtsubo et al. 2012). Gaps were closed using polymerase chain reaction and Sanger sequencing.

### Annotation and Structural Analysis of the Genomes

Initial gene predictions and annotations were conducted using DFast pipeline (Tanizawa et al. 2018), followed by manual corrections with the aid of RNAmmer 1.2 Server (Lagesen et al. 2007), the National Center for Biotechnology Information (NCBI) ORFfinder (Wheeler et al. 2003), and BLAST searches. Functional annotation of the predicted genes was conducted using EggNOG 4.5.1 (Huerta-Cepas et al. 2017). Protein structures were analyzed using NCBI Conserved Domain Database (CDD) (Lu et al. 2020), Pfam (El-gebali et al. 2019), and PROSITE (Sigrist et al. 2013). Metabolic pathways were analyzed using Kyoto Encyclopedia of Genes and Genomes (KEGG) (Kanehisa 2019) and UniProt Knowledgebase (UniProtKB) (UniProt Consortium 2019). Dinucleotide bias and GC skew were analyzed using ArcWithColor 1.47 (Ohtsubo et al. 2008). The codon adaptation index (CAI) was calculated using the CAIcal server (Puigbo et al. 2008). Pairwise comparisons between the symbiont genomes from *D*. cf *continua* and *D. citri* were performed using GenomeMatcher 2.3 (Ohtsubo et al. 2008), in which BlastN of all-against-all bl2seq similarity searches was conducted with the parameter set “-F F -W 21 -e 1.0e-10.”

### Substitution Rate Analysis

Amino acid sequences were deduced from the protein-coding sequences (CDSs) shared between the symbiont genomes from *D*. cf. *continua* and *D*. *citri* and then aligned with MAFFT 7.452 (Katoh and Standley 2013) using the E-INS-i algorithm with default parameters. The resulting protein alignments were converted to nucleotide alignments using PAL2NAL 13.0 (Suyama et al. 2006). Nonsynonymous (d*N*) and synonymous (d*S*) substitution rates and d*N*/d*S* ratios between orthologous pairs were calculated using the KaKs_Calculator 1.2 package (Zhang et al. 2006) implementing the YN model (Yang and Nielsen 2000). All statistical analyses were performed using the R software version 3.6.3 (R Core Team 2020, https://www.r-project.org; last accessed August 24, 2020).

### Analysis of Polyketide Synthase Genes

The domain architecture and function of polyketide synthase (PKS) gene products were derived by analyses with the *trans*-acyltransferase (AT) PKS annotation and structure prediction tool TransATor (Helfrich et al. 2019) (https://transator.ethz.ch/; last accessed August 24, 2020), protein alignments with the pederin PKS using the MUSCLE algorithm (Edgar 2004) as implemented in Geneious 8 (https://www.geneious.com/; last accessed August 24, 2020), and NCBI BLAST searches.

### Phylogenetic Analysis

Protein sequences were aligned with MAFFT 7.452 (Katoh and Standley 2013) using the E-INS-i algorithm with default parameters. Amino acid sites corresponding to alignment gap(s) were omitted from the data set. Phylogenetic trees were inferred by the maximum likelihood method using RAxML 8.2.12 (Stamatakis 2014) with 1,000 replicates using the WAG (Whelan and Goldman) matrix of the amino acid replacements, assuming a proportion of invariant positions and four gamma-distributed rates (WAG+I+gamma model). Taxonomic assignment of bacteria was based on the Genome Taxonomy Database (GTDB), in which the former class Betaproteobacteria is reclassified as Burkholderiales, an order within the class Gammaproteobacteria (Parks et al. 2018).

## Results and Discussion

### Basic Features of the *Profftella*_Dco Genome

**Fig. 1.**
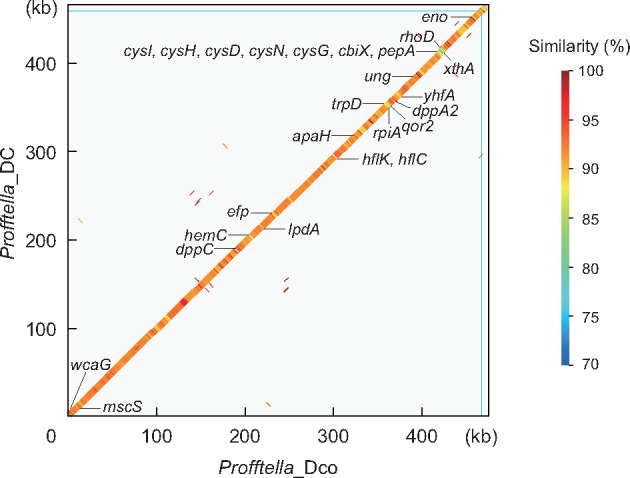
Comparison of the genomic structures of *Profftella*_Dco and *Profftella*_DC. The genomes of *Profftella*_Dco and *Profftella*_DC are represented by the *x* and *y* axes, respectively. The thick line indicates the shared synteny between the two genomes. The color of the line indicates the percentage similarity between the nucleotide sequences. For each genome, the chromosome and the plasmid were concatenated, the borders of which are indicated by thin blue lines. The genes found in *Profftella*_Dco, but not in *Profftella*_DC, are presented below the line plot; the genes present in *Profftella*_DC, but not in *Profftella*_Dco, are shown above the line plot.

The analysis using a total of 716 Mb in 1.54 million MiSeq reads followed by Sanger sequencing identified the complete chromosome sequence of *Profftella*_Dco consisting of 469,264 bp (24.4% GC), and its plasmid sequence consisting of 5,952 bp (23.1% GC) with a single gap (table 1, supplementary fig. S1, Supplementary Material online). The gap left in the plasmid corresponds to an ∼900-bp region encoding two hypothetical proteins in the plasmid of *Profftella*_DC (Nakabachi, Ueoka, et al. 2013). This nearly complete genome encodes 355 predicted protein-coding sequences (CDSs), 10 putative pseudogenes, a single rRNA operon, and 34 tRNAs (table 1, supplementary table S1, Supplementary Material online). A genome-wide alignment of homologous nucleotide sequences in *Profftella*_Dco and *Profftella*_DC revealed that these genomes retain highly conserved synteny (fig. 1), indicating that most genes are shared between *Profftella*_Dco and *Profftella*_DC, and essentially no genome rearrangements have occurred since they diverged. Between these genomes, a total of 377 pairs of orthologous genes are 91.7% identical on average at the nucleotide level, and amino acid sequences of 341 pairs of orthologous CDSs are 90.0% identical on average. *Profftella*_Dco and *Profftella*_DC share all the genes involved in the biosynthesis of diaphorin, hemolysin, riboflavin, biotin, and carotenoids, which will be further discussed later.

**Table 1:** Genomic Features of *Profftella* and *Carsonella* from *Diaphorina* cf. *continua* and *Diaphorina citri*

	*Profftella*_Dco	*Profftella*_DC	*Carsonella*_Dco	*Carsonella*_DC
Chromosome size (bp)	469,264	459,399	173,853	174,014
Plasmid size (bp)	>5,952	5,458	—	—
G+C content [plasmid] (%)	24.4 [23.1]	24.2 [23.9]	17.9	17.6
CDS [plasmid]	355 [4]	365 [6]	202	207
rRNA	3	3	3	3
tRNA	34	34	28	27

**Fig. 2.**
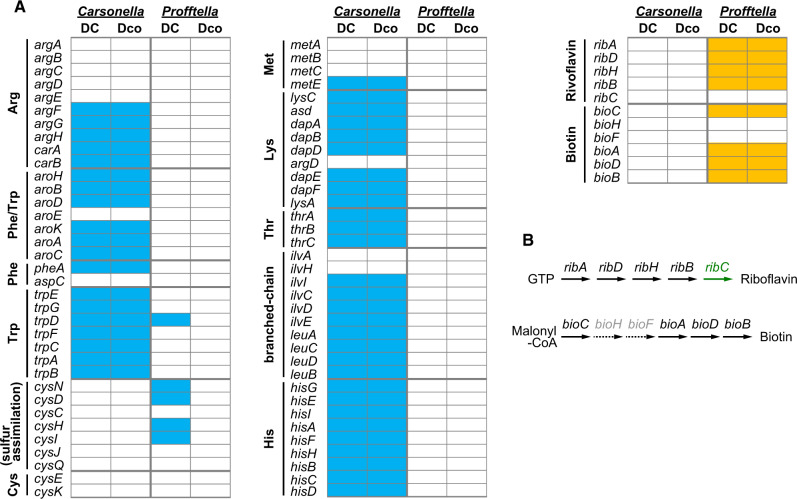
The repertoire of *Carsonella* and *Profftella* genes that are potentially involved in providing nutrition. (*A*) Amino acid and vitamin biosynthetic genes in the genomes of *Carsonella* and *Profftella* from *Diaphorina* cf. *continua* and *Diaphorina citri*. Filled and open boxes indicate the presence and absence of genes, respectively. (*B*) B vitamin synthetic pathways reconstructed from genes retained in the genomes of *Profftella*_Dco and *Profftella*_DC. Genes shown in black type are found in the *Profftella* genomes. Those in gray type appear to be absent in the genomes. The *ribC* shown in green type is a host psyllid gene that was horizontally acquired from an unknown bacterium.

Despite this high level of conservation between the genomes, random gene silencing appears to be still ongoing in *Profftella*_Dco and *Profftella*_DC. The genes found in one of the *Profftella* lineages but not in the other are shown in figure 1 and supplementary table S2, Supplementary Material online. BLAST searches and phylogenetic analyses of these genes revealed no sign of horizontal acquisition following the divergence of these symbionts, indicating that the different gene sets reflect gene silencing on either genome. The functional categories of these genes vary (supplementary table S2, Supplementary Material online), implying that the gene silencing has been randomly occurring. However, it may be notable that the genes for sulfur assimilation (*cysDHIN*) are retained in *Profftella*_DC but are lost in *Profftella*_Dco (figs. 1 and 2*A*). These genes could potentially contribute to the synthesis of sulfur-containing amino acids, cysteine and methionine, the latter of which is an essential amino acid that the host psyllids are unable to synthesize. However, no other genes related to amino acid synthesis are retained in the *Profftella* genome. Moreover, *Carsonella*_Dco and *Carsonella*_DC lack genes for the biosynthesis of cysteine/methionine other than *metE*, which converts homocysteine into methionine (fig. 2*A*). Therefore, the cysteine/methionine synthesis pathway appears to be incomplete, even with the aid of the host psyllids (Sloan et al. 2014), thus making the role of the conservation of *cysDHIN* genes in *Profftella*_DC unclear.

### Polyketide Biosynthesis in *Profftella*_Dco

**Fig. 3.**
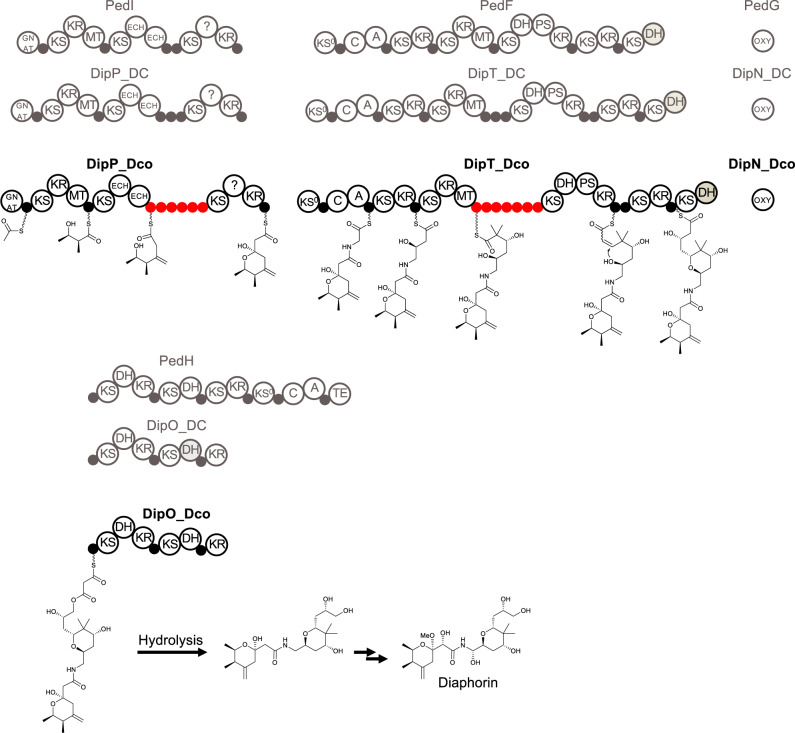
Architecture of the PKS proteins from *Profftella*_Dco (Dip_Dco, shown in bold), with the predicted biosynthetic pathway for diaphorin in *Profftella*_Dco. For comparison, PKS orthologs from *Profftella*_DC (Dip_DC) and the *Paederus* symbiont (Ped) are shown in gray, above Dip_Dcos. A, nonribosomal peptide synthetase (NRPS) adenylation domain; C, NRPS condensation domain; DH, dehydratase; GNAT, GCN5-related *N*-acetyltransferase superfamily (usually serving as acetyltransferase in PKSs); ECH, enoyl-CoA reductase-like domain; KR, ketoreductase; KS, ketosynthase; KS^0^, nonelongating KS; MT, C-methyltransferase; PS, putative pyrane synthase; OXY, oxygenase; TE, thioesterase; ?, region of unknown function. The domains in gray are predicted to be inactive due to missing active-site residues. The small circles denote carrier protein domains including ACPs and PCPs (NRPS peptidyl carrier proteins). Unusually amplified ACPs in DipP_Dco and DipT_Dco are highlighted in red (see also supplementary text S1, Supplementary Material online).

Twenty genes (*dipA*-*T*) constituting the PKS biosynthetic gene clusters are perfectly conserved in *Profftella*_Dco, manifesting that polyketide synthesis is an important function of the *Profftella* lineages (supplementary fig. S1*A* and table S1, Supplementary Material online). As in *Profftella*_DC (Nakabachi, Ueoka, et al. 2013), the PKS system is separated into two loci within the *Profftella*_Dco chromosome (supplementary fig. S1*A*, Supplementary Material online). These loci correspond to 15.4% (72,207/469,264 bp) of the chromosome. The genes from *Profftella*_Dco and *Profftella*_DC form highly supported clades in the constructed phylogenetic tree (supplementary fig. S2, Supplementary Material online), and domain structures of their encoded proteins are also perfectly conserved, except that acyl carrier proteins (ACPs) are extensively amplified in DipP and DipT of *Profftella*_Dco (fig. 3, supplementary text S1, Supplementary Material online). DipP, which is orthologous to PedI of the *Pseudomonas* (Gammaproteobacteria: Pseudomonadales) symbiont of *Paederus* rove beetles (Coleoptera: Staphylinidae) (Piel et al. 2004), is an enzyme with four modules that catalyzes the initiation of the synthesis, and three steps of the extension, of the polyketide chain (fig. 3). DipT, which is orthologous to PedF of the *Paederus* symbiont (Piel et al. 2004), is an enzyme with seven modules that assembles the largest part of the diaphorin molecule by catalyzing the extension and modification of the polyketide chain received from DipP (fig. 3). The *dip* and *ped* PKS belong to the *trans*-AT PKS family, in which the recombination of module-encoding gene regions is a common mechanism of metabolic diversification (Nguyen et al. 2008; Ueoka et al. 2015). However, an analysis with the prediction tool TransATor (Helfrich et al. 2019), which proposes polyketide structures based on ketosynthase domains in *trans*-AT PKSs, demonstrated that both *Profftella* pathways generate the same compound diaphorin (fig. 3).

Whereas PedI has two ACP domains between the enoyl-CoA reductase-like domain and the ketosynthase domain, DipP of *Profftella*_DC (DipP_DC) has three ACPs at the corresponding site. Furthermore, there are as many as six ACP domains at the corresponding site of DipP from *Profftella*_Dco (DipP_Dco) (fig. 3, supplementary text S1, Supplementary Material online). Similarly, whereas PedF has only a single ACP domain between the C-methyltransferase and ketosynthase domains, DipT_DC has three, and DipT_Dco has as many as seven ACP domains at the corresponding site (fig. 3, supplementary text S1, Supplementary Material online). Large ACP series also occurs in the PKS-like monomodular polyunsaturated fatty acid synthases (Jiang et al. 2008; Hayashi et al. 2016), and ACP pairs or triplets have been identified in some *trans*-AT PKS modules (Gu et al. 2011; Helfrich and Piel 2016). However, to our knowledge, extremely amplified ACPs in modular PKSs like these are not known from any other organism. It is shown for polyunsaturated fatty acid synthases as well as trans-AT PKSs that the production titers can correlate with ACP numbers in repeat regions (Jiang et al. 2008; Gu et al. 2011; Hayashi et al. 2016; Helfrich and Piel 2016). The amplification of ACP domains in the *Profftella*_Dco as compared with *Profftella*_DC may therefore improve the efficiency of diaphorin synthesis. It would be interesting to assess this possibility in future studies.

### Another Gene Potentially Related to Toxicity

**Fig. 4.**
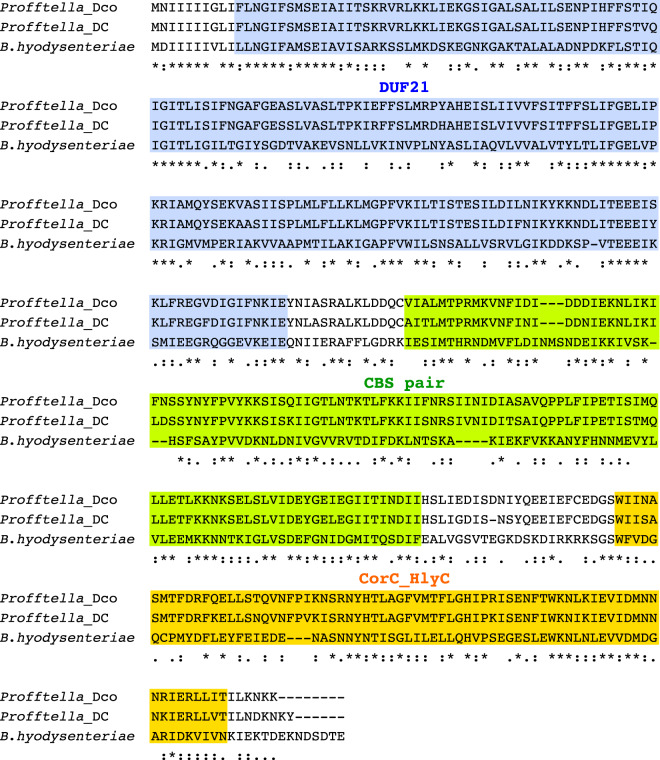
Amino acid sequences of TlyC orthologs from *Profftella*_Dco and *Profftella*_DC, which are aligned with that from *Brachyspira hyodysenteriae*, whose membranolytic activity has been experimentally confirmed (ter Huurne et al. 1994). Sequences are aligned with MAFFT 7.452 using the E-INS-i algorithm with default parameters. Identical, conservative, and semiconservative residues are marked with asterisks, double dots, and single dots, respectively. DUF21, CBS pair, and CorC_HlyC domains are indicated with blue, green, and orange shades, respectively. Conserved domain structures are based on information of the NCBI Conserved Domain Database implemented with models imported from external databases, including Pfam, SMART, and COG (Lu et al. 2020).

*Profftella*_Dco and *Profftela*_DC are shown to have *tlyC* ortholog encoding a protein with the CorC_HlyC domain, the DUF21 domain, and two tandem repeats of the cystathionine beta-synthase (CBS pair) domains (fig. 4). The set of these domains constitutes the characteristic feature of the TlyC superfamily of hemolysin and related proteins (Bateman 1997). Hemolysins are bacterial toxins that cause lysis of red blood cells by destroying their cell membrane, and the TlyC (hemolysin C) of *Brachyspira hyodysenteriae* (Spirochaetota), a causative agent of swine dysentery, was first found to exhibit hemolytic and cytotoxic activities (ter Huurne et al. 1994). Subsequently, similar activities including disruption of cytoplasmic and intracellular membranes were observed for TlyC orthologs of typhus pathogens, *Rickettsia typhi* (Alphaproteobacteria) and *Rickettsia prowazekii* (Radulovic et al. 1999; Whitworth et al. 2005). These findings suggest that TlyCs interact with the host cell membrane and rupture it although its mechanism is not defined (ter Huurne et al. 1994; Radulovic et al. 1999; Whitworth et al. 2005). The TlyCs of *Profftella_*Dco and *Profftella*_DC are 34.6% and 32.8% identical, respectively, to that of *B. hyodysenteriae* (fig. 4). Therefore, they may also have membranolytic activity, which could potentially function as a defensive toxin, like diaphorin, to protect the holobiont (host + symbionts) from natural enemies. Another possibility is that the TlyCs are involved in manipulating the host membrane, for example, at the process of escaping from a large central syncytium of the bacteriome and entering into the oocytes during transovarial transmission (Dan et al. 2017).

### Carotenoid Biosynthesis in *Profftella*

**Fig. 5.**
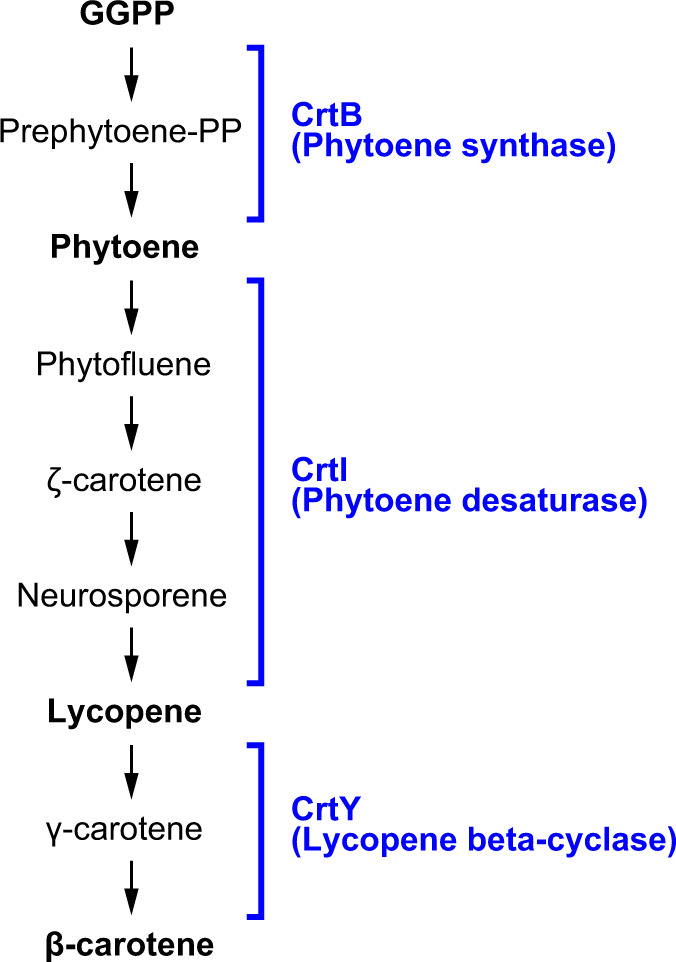
Biosynthetic pathway of carotenoids, the steps of which are catalyzed by CrtB (phytoene synthase), CrtI (phytoene desaturase), and CrtY (lycopene beta-cyclase). GGPP, geranylgeranyl diphosphate.

The plasmid of *Profftella*_Dco encodes genes for biosynthesis of carotenoids (*crtB*, *crtI*, and *crtY*) (table 2, fig. 5, and supplementary fig. S1B, Supplementary Material online), which are also found in the plasmid of *Profftella*_DC (Nakabachi, Ueoka, et al. 2013). As shown in figure 5, *crtB* encodes phytoene synthase (EC:2.5.1.32), which catalyzes the condensation of two molecules of geranylgeranyl diphosphate to give prephytoene diphosphate and the subsequent rearrangement of the cyclopropylcarbinyl intermediate to yield the 15-*cis*-phytoene isomer (Fraser and Bramley 2004). The *crtI* gene encodes phytoene desaturase (EC:1.3.99.31), which converts 15-*cis*-phytoene into all-*trans*-lycopene via the intermediates phytofluene, zeta-carotene, and neurosporene, through the introduction of four double bonds (Fraser and Bramley 2004). The *crtY* gene encodes lycopene beta-cyclase (EC:5.5.1.19), which catalyzes the cyclization of both ends of lycopene to form beta-carotene (fig. 5). Carotenoids are yellow, orange, and red organic pigments that are widely distributed in diverse lineages of organisms on Earth (Fraser and Bramley 2004; Mussagy et al. 2019). In animals, these compounds play important roles, including antioxidation and pigmentation for photoprotection, camouflage, or ornamentation (Fraser and Bramley 2004; Mussagy et al. 2019). They are synthesized by various lineages of bacteria, archaea, protists, fungi, and plants, but animals generally lack the ability to produce carotenoids and must obtain them through their diet. Unique exceptions are aphids and mites, which have horizontally acquired carotenoid biosynthesis genes from fungi (Moran and Jarvik 2010; Altincicek et al. 2012). In addition, the chromosome of *Candidatus* Portiera aleyrodidarum (Gammaproteobacteria: Oceanospirillales), the primary symbiont of whiteflies (Hemiptera: Aleyrodoidea), is shown to encode *crtB*, *crtI*, and *crtY* (Santos-Garcia et al. 2012; Sloan and Moran 2012a, 2013), which are the same set of gene orthologs found in the *Profftella* lineages. The gene sets encoded in the genomes of *Profftella* and *Portiera* appear to be of bacterial origin and independent of each other (table 2, supplementary fig. S3, Supplementary Material online). The plasmids of *Profftella* may have been horizontally acquired from other bacterial lineages, but neither phylogenetic analysis of CrtI encoded therein (supplementary fig. S3, Supplementary Material online), GC content (23.1% vs. 24.4% of the chromosome), nor CAI (0.769 ± 0.011 vs. 0.774 ± 0.002 of the chromosome) shows clear evidence of recent horizontal acquisition. In any case, conservation of the carotenoid biosynthetic plasmid in both *Profftella*_Dco and *Profftella*_DC indicates that carotenoid synthesis is a crucial function for *Profftella*.

**Table 2:** Carotenoid Biosynthesis Genes in the *Profftella*_Dco Genome

Gene ID	Product	Length (aa)	Top BLAST Hit	Source Organism	*E*-value	Identity (aa %)
*crtB*	Phytoene synthase	330	WP_105077524	*Pantoea ananatis* (Gammaproteobacteria)	4E-96	45.8
*crtI*	Phytoene desaturase	493	WP_154058492	*Pseudescherichia vulneris* (Gammaproteobacteria)	0	57.8
*crtY*	Lycopene beta-cyclase	392	GAL56634	*Pseudescherichia vulneris* (Gammaproteobacteria)	3E-117	44.4

Previous studies have suggested sister relationships not only between whiteflies and psyllids but also between their primary symbionts *Portiera* and *Carsonella* (Spaulding and von Dohlen 1998; Thao and Baumann 2004; Sloan and Moran 2012a). Therefore, the common ancestor of *Portiera* and *Carsonella* may have possessed carotenoid biosynthesis genes. However, all *Carsonella* lineages sequenced to date including *Carsonella*_Dco (supplementary table S3, Supplementary Material online) and *Carsonella*_DC lack them (Nakabachi et al. 2006; Sloan and Moran 2012b; Nakabachi, Ueoka, et al. 2013). Thus, the gene set found in the *Profftella* lineage appears to add another important role to this secondary symbiont. Future studies should focus on the analysis of carotenoid products and their functional roles in *Diaphorina* psyllids.

### Nutritional Contribution of *Profftella*

Sixteen genes for “coenzyme transport and metabolism (COG category H)” found in *Profftella*_DC are perfectly conserved in *Profftell*a_Dco (supplementary table S1, Supplementary Material online). The genes in this category include those for the synthesis of riboflavin (*ribA*, *ribB*, *ribD*, and *ribH*) and biotin (*bioA*, *bioB*, *bioC*, and *bioD*) (fig. 2). Although both *Profftella* lineages lack *ribC* that is required for the final step of riboflavin synthesis, a previous study demonstrated that host psyllids possess *ribC* that has been horizontally acquired from a bacterium (Sloan et al. 2014), which can potentially complete the biosynthetic pathway of riboflavin (fig. 2*B*). On the other hand, the pathway for biotin synthesis appears to be incomplete in the psyllid–*Profftella*–*Carsonella* symbiotic system (fig. 2*B*). Still, besides the *bio* genes, the *Profftella* lineages possess the *birA* gene encoding a biotin–[acetyl-CoA-carboxylase] ligase, which is involved in the metabolism of biotin (supplementary table S1, Supplementary Material online). This pattern of gene conservation implies that biosynthesis and metabolism of biotin are important processes in *Profftella*.

These genes mentioned above are essentially the only contributing factors to *Profftella*’s role as a nutritional symbiont, which possibly play a crucial role in stabilizing symbiotic relationships between the host and this unique organelle-like defensive symbiont.

### Features of the *Carsonella*_Dco Genome

A total of 0.15 million MiSeq reads yielding 38 Mb were assembled into the complete *Carsonella*_Dco genome consisting of a single circular chromosome of 173,853 bp with a GC content of 17.9% (table 1, supplementary fig. S4*A*, Supplementary Material online). It encodes 202 CDSs, a single putative pseudogene, a single rRNA operon, and 28 tRNAs (table 1, supplementary table S3, Supplementary Material online). The whole-genome alignment of *Carsonella*_Dco and *Carsonella*_DC (CP003467.1) revealed a strong colinearity between these genomes (supplementary fig. S4*B*, Supplementary Material online), indicating that there have been no genome rearrangements since the divergence of these symbiont lineages. Similar colinear patterns have also been observed among other *Carsonella* genomes (Sloan and Moran 2012b; Nakabachi, Ueoka, et al. 2013), as well as in the genomes of other bacteriome-associated primary symbionts (Tamas et al. 2002; Degnan et al. 2005; Rio et al. 2012; Bennett and Moran 2013; Koga and Moran 2014; Husnik and McCutcheon 2016; Mao et al. 2017). All genes with functional annotations are shared between *Carsonella*_Dco and *Carsonella*_DC (supplementary table S3, Supplementary Material online). In these genomes, a total of 233 pairs of orthologous genes exhibited 91.9% nucleotide identity on average, and 202 pairs of orthologous CDSs exhibited 89.6% amino acid identity on average.

As previously shown for *Carsonella*_DC (Nakabachi, Ueoka, et al. 2013), the gene inventory of *Carsonella*_Dco (fig. 2*A*) indicates that this symbiont is a typical nutritional symbiont that provides essential amino acids to its host psyllid. *Carsonella*_Dco and *Carsonella*_DC share seven genes (*trpA*–*G*) constituting the intact tryptophan biosynthesis pathway, and nine genes (*hisA*–*I*) encoding the intact histidine biosynthesis pathway, whereas the other *Carsonella* lineages analyzed to date partially or completely lack these genes (Nakabachi et al. 2006; Sloan and Moran 2012b). In two psyllid species *Ctenarytaina eucalypti* (Aphalaridae) and *Heteropsylla cubana* (Psyllidae), missing genes are complemented by secondary symbionts (Gammaproteobacteria: Enterobacterales) that are distantly related to *Profftella* (Sloan and Moran 2012b). The conservation of the biosynthetic pathways of tryptophan and histidine in *Carsonella*_Dco and *Carsonella*_DC indicates the broader metabolic capacities of these primary symbionts and highlights the limited nutritional capacity of *Profftella*.

### Genome-Wide Substitution Rates in *Profftella* and *Carsonella*

**Fig. 6.**
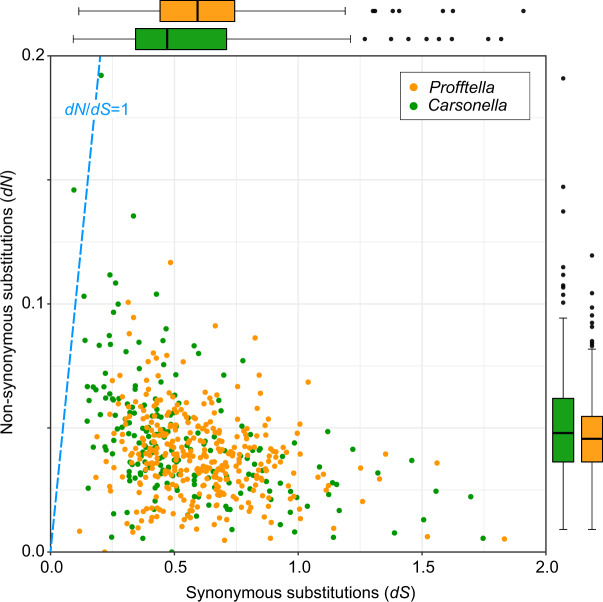
Synonymous (d*S*) and nonsynonymous (d*N*) substitution rates inferred from pairwise comparisons of orthologous CDSs in lineages of *Profftella* (orange dots) and *Carsonella* (green dots). A total of 341 and 202 orthologous pairs in *Profftella* and *Carsonella*, respectively, were used for the analysis. Box plots (*Profftella*, orange; *Carsonella*, green) on the *x* and *y* axes indicate distributions (median, quartiles, minimum, maximum, and outliers) of d*S* and d*N* values, respectively.

Whereas various lineages of primary symbionts have been repeatedly shown to have highly stable genomic structures (Tamas et al. 2002; Degnan et al. 2005; Rio et al. 2012; Sloan and Moran 2012b; Bennett and Moran 2013; Nakabachi, Ueoka, et al. 2013; Koga and Moran 2014; Husnik and McCutcheon 2016; Mao et al. 2017), this is often not the case for secondary or more recently acquired symbionts (Burke and Moran 2011; Manzano-marin et al. 2012; Bennett et al. 2014; Koga and Moran 2014; Campbell et al. 2015; McCutcheon et al. 2019). However, as mentioned above, the synteny analysis revealed that not only *Carsonella* but also *Profftella* retain highly conserved genomic structures (fig. 1 and supplementary fig. S4*B*, Supplementary Material online). This highly conserved synteny in *Profftella* may be indicative of genomic stasis in this symbiont and may simply reflect a short divergence time. Thus, to evaluate the stability of these genomes, we further analyzed genome-wide rates of synonymous (d*S*) and nonsynonymous (d*N*) substitutions for *Profftella* and *Carsonella*, for which the same divergence time can be applied (fig. 6, supplementary tables S1 and S3, Supplementary Material online). The average d*S* rate was higher (Mann–Whitney *U* test, *P *<* *0.001) in *Profftella* (0.605 ± 0.013) than in *Carsonella* (0.545 ± 0.022). No genes appeared to be at or near saturation for d*S* (>2.0). On the other hand, the average d*N* was higher (Mann–Whitney *U* test, *P *<* *0.05) in *Carsonella* (0.045 ± 0.002) than in *Profftella* (0.039 ± 0.001). Accordingly, the average d*N*/d*S* ratio was higher (Mann–Whitney *U* test, *P *<* *0.001) in *Carsonella* (0.130 ± 0.011) than in *Profftella* (0.076 ± 0.003). With a single exception of a *Carsonella* gene encoding a hypothetical protein, all genes were estimated to have d*N*/d*S* <1, indicating purifying selection for most genes in these symbionts. Whereas the differences in d*S* and d*N* between *Profftella* and *Carsonella* were statistically significant, they were much less than double, indicating that *Profftella* and *Carsonella* have a similar level of nucleotide substitutions (fig. 6). This is a stark contrast to the cases of *Ca*. Sulcia muelleri (Bacteroidota) and various lineages of coresident symbionts in which DNA substitution rates are 1–2 orders of magnitude higher in more recently acquired coresidents than in *Sulcia* (Bennett et al. 2014; Campbell et al. 2015; Silva and Santos-Garcia 2015). The highly conserved synteny and substitution rates that are similar to those of *Carsonella* imply that *Profftella* has acquired a relatively stable status, which is comparable to that of obligate primary symbionts.

## Conclusions

The present study revealed that *Profftella*_Dco and *Profftella*_DC share all the genes involved in the biosynthesis of diaphorin, hemolysin, riboflavin, biotin, and carotenoids, underlining multiple roles of *Profftella*, which may contribute to the stabilization of symbiotic relationships with their host psyllids. However, ACPs were extensively amplified in enzymes to synthesize diaphorin in *Profftella*_Dco. This ACP augmentation, which is unprecedented in modular PKSs of any organism, is not thought to influence the polyketide structure but may improve the efficiency of synthesis. Analyses of synteny and genome-wide substitution rates revealed that the *Profftella* genome is stable similarly to the *Carsonella* genome, implying that *Profftella* has acquired the status that is comparable to that of primary nutritional symbionts.

## Supplementary Material

Supplementary data are available at *Genome Biology and Evolution* online.

## Supplementary Material

evaa175_Supplementary_Data

## Notes

**Data deposition:** This project has been deposited at DDBJ/EMBL/GenBank databases under the accession numbers AP023215 (*Profftella*_Dco chromosome), AP023216 (*Profftella*_Dco plasmid), and AP023214 (*Carsonella*_Dco).
