# Supplementary material for: Comparative Genomics Underlines Multiple Roles of Profftella, an Obligate Symbiont of Psyllids: Providing Toxins, Vitamins, and Carotenoids
Source: Genome Biol Evol. 2020 Aug 14;12(11):1975–87. doi: 10.1093/gbe/evaa175 (PMC7643613; doi:10.1093/gbe/evaa175)
Supplement: evaa175_Supplementary_Data [file evaa175_supplementary_data.zip › FigS1_Profftella_genome_200613.pdf]

A

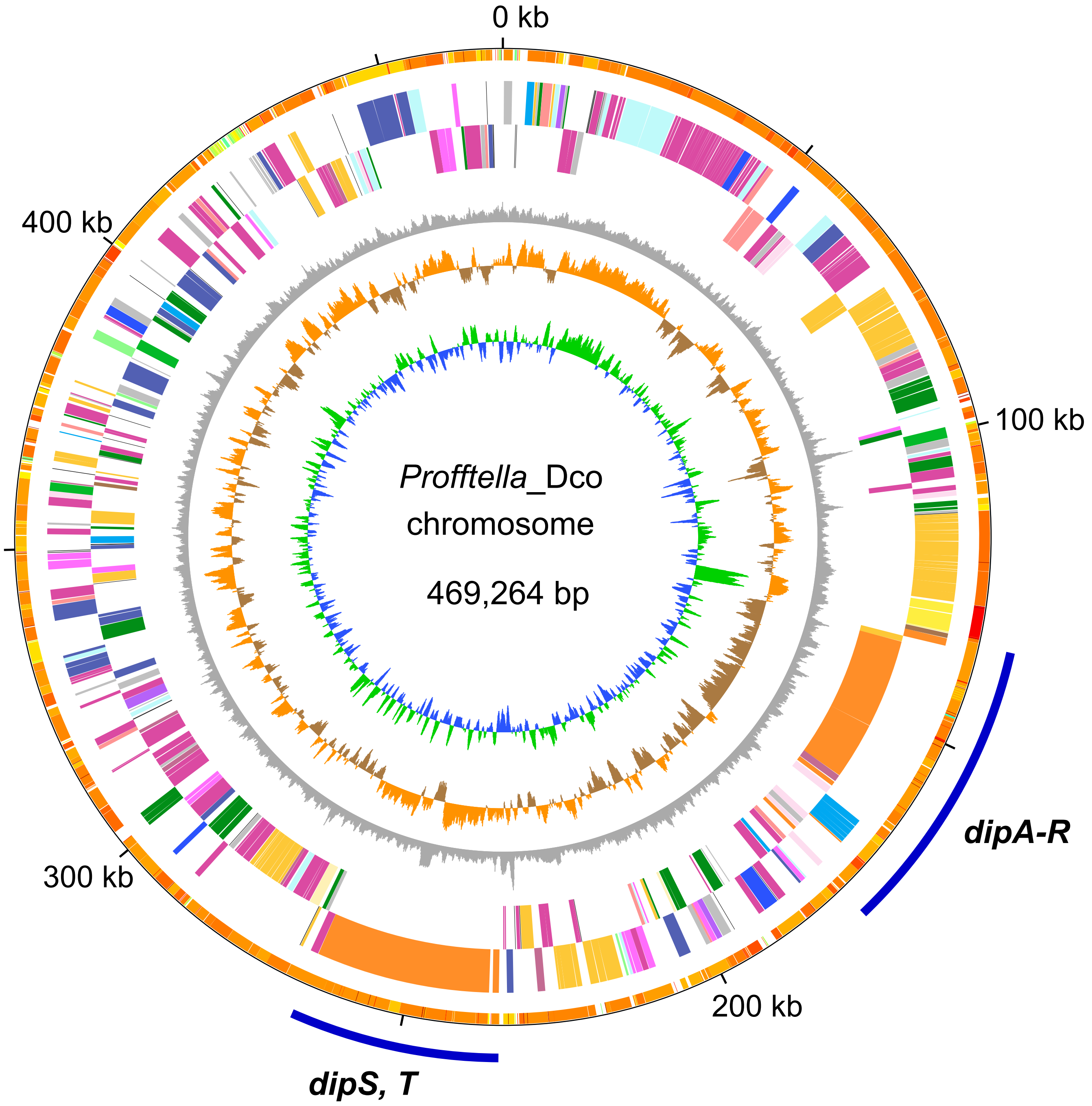

0 kb
100 kb
200 kb
300 kb
400 kb
*Profftella*_Dco
chromosome
469,264 bp
dipA-R
dipS, T

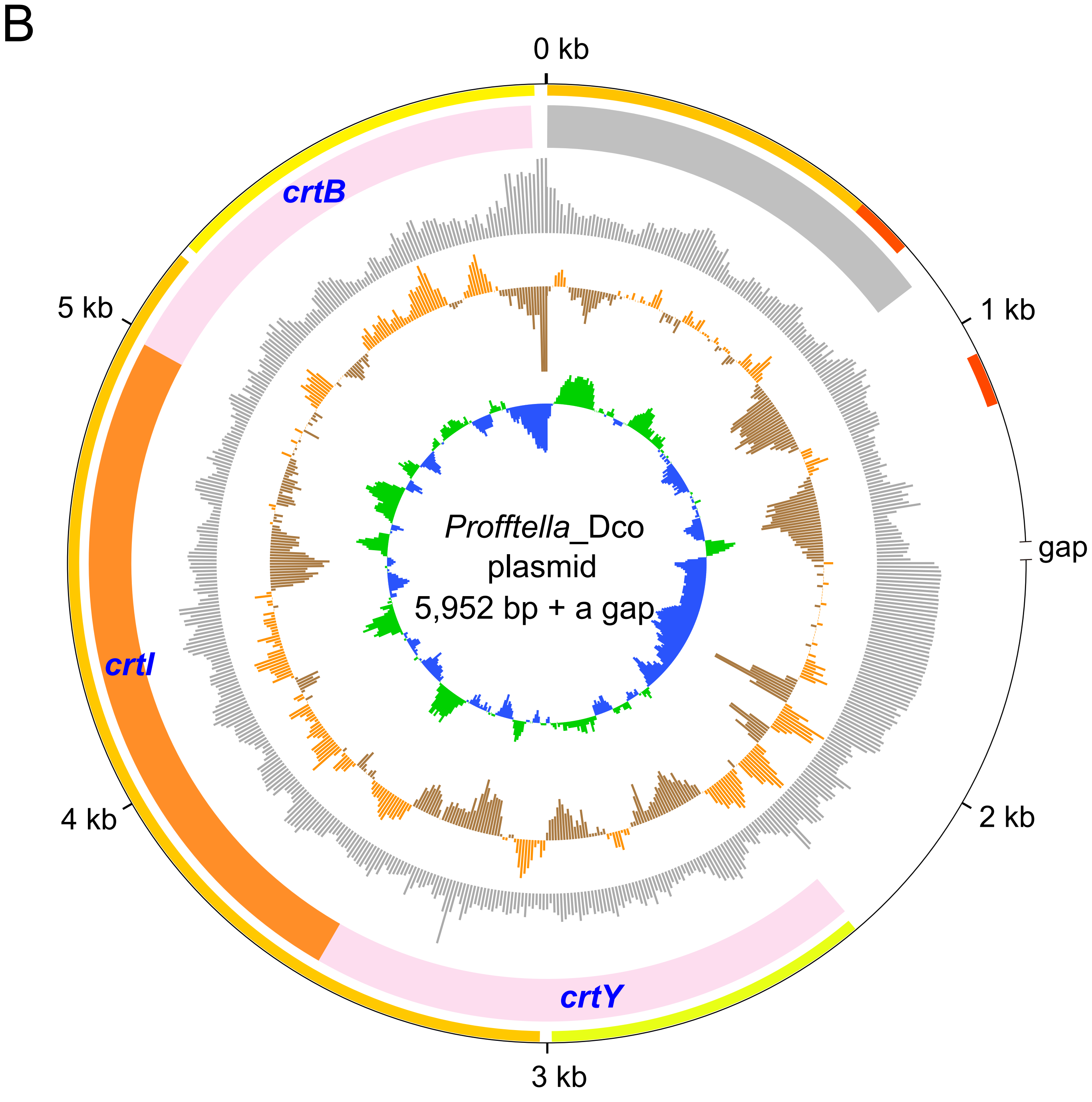

B
0 kb
1 kb
gap
2 kb
3 kb
4 kb
5 kb
crtB
crtI
crtY
*Profftella*_Dco
plasmid
5,952 bp + a gap

Color scale for (ii) sequence similarity to the corresponding symbiont genome in *D. citri*.

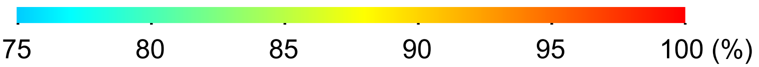

Functional categories of (iii, iv) genes encoded by *Profftella*_Dco and *Carsonella*_Dco.

- [Q] Secondary metabolites biosynthesis, transport and catabolism
- [L] DNA replication, recombination and repair
- [K] Transcription
- [J] Translation, ribosomal structure and biogenesis
- [O] Posttranslational modification, protein turnover, chaperones
- [C] Energy production and conversion
- [G] Carbohydrate transport and metabolism
- [I] Lipid transport and metabolism
- [F] Nucleotide transport and metabolism
- [E] Amino acid transport and metabolism
- [H] Coenzyme transport and metabolism
- [P] Inorganic ion transport and metabolism
- [M] Cell wall/membrane/envelope biogenesis
- [D] Cell cycle control, cell division, chromosome partitioning
- [N] Cell Motility
- [U] Intracellular trafficking, secretion, and vesicular transport
- [T] Signal transduction mechanisms
- [R] General function prediction only / [S] Function unknown / Not in COGs
- rRNA
- tRNA

Fig. S1.- Circular representation of the genome of *Profftella*_Dco. (A) Chromosome. The dip loci are highlighted. (B) Plasmid. Carotenoid biosynthesis genes are indicated. The concentric rings denote the following features (from the outside): (i) the scale in kilobases, (ii) nucleotide similarity to the *Profftella*_DC genome, (iii) forward strand genes, (iv) reverse strand genes, (v) dinucleotide bias, (vi) GC skew, and (vii) G + C content.
For the calculation of (v), (vi), and (vii), sliding windows of 1,000 bp and a step size of 100 bp were used for the chromosome, whereas 100 bp windows and a step size of 10 bp were used for the plasmid.
