# Supplementary material for: Comparative Genomics Underlines Multiple Roles of Profftella, an Obligate Symbiont of Psyllids: Providing Toxins, Vitamins, and Carotenoids
Source: Genome Biol Evol. 2020 Aug 14;12(11):1975–87. doi: 10.1093/gbe/evaa175 (PMC7643613; doi:10.1093/gbe/evaa175)
Supplement: evaa175_Supplementary_Data [file evaa175_supplementary_data.zip › FigS2_Dip_tree_200613.pdf]

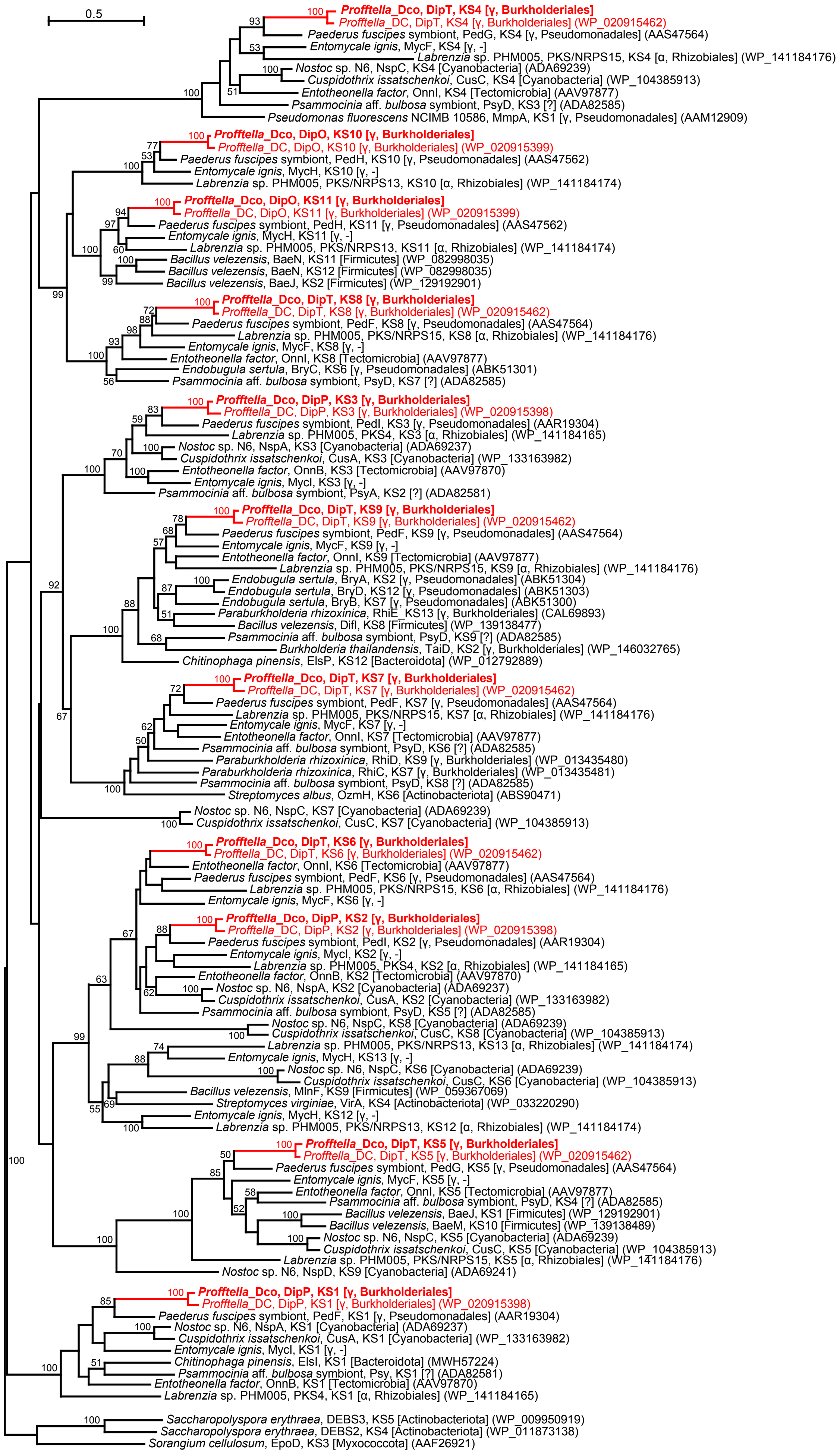

0.5
Profftella_Dco, DipT, KS4 [γ, Burkholderiales]
Profftella_DC, DipT, KS4 [γ, Burkholderiales] (WP_020915462)
Paederus fuscipes symbiont, PedG, KS4 [γ, Pseudomonadales] (AAS47564)
Entomycale ignis, MycF, KS4 [γ, -]
Labrenzia sp. PHM005, PKS/NRPS15, KS4 [α, Rhizobiales] (WP_141184176)
Nostoc sp. N6, NspC, KS4 [Cyanobacteria] (ADA69239)
Cuspidothrix issatschenkoi, CusC, KS4 [Cyanobacteria] (WP_104385913)
Entotheonella factor, OnnI, KS4 [Tectomicrobia] (AAV97877)
Psammocinia aff. bulbosa symbiont, PsyD, KS3 [?] (ADA82585)
Pseudomonas fluorescens NCIMB 10586, MmpA, KS1 [γ, Pseudomonadales] (AAM12909)
Profftella_Dco, DipO, KS10 [γ, Burkholderiales]
Profftella_DC, DipO, KS10 [γ, Burkholderiales] (WP_020915399)
Paederus fuscipes symbiont, PedH, KS10 [γ, Pseudomonadales] (AAS47562)
Entomycale ignis, MycH, KS10 [γ, -]
Labrenzia sp. PHM005, PKS/NRPS13, KS10 [α, Rhizobiales] (WP_141184174)
Profftella_Dco, DipO, KS11 [γ, Burkholderiales]
Profftella_DC, DipO, KS11 [γ, Burkholderiales] (WP_020915399)
Paederus fuscipes symbiont, PedH, KS11 [γ, Pseudomonadales] (AAS47562)
Entomycale ignis, MycH, KS11 [γ, -]
Labrenzia sp. PHM005, PKS/NRPS13, KS11 [α, Rhizobiales] (WP_141184174)
Bacillus velezensis, BaeN, KS11 [Firmicutes] (WP_082998035)
Bacillus velezensis, BaeN, KS12 [Firmicutes] (WP_082998035)
Bacillus velezensis, BaeJ, KS2 [Firmicutes] (WP_129192901)
Profftella_Dco, DipT, KS8 [γ, Burkholderiales]
Profftella_DC, DipT, KS8 [γ, Burkholderiales] (WP_020915462)
Paederus fuscipes symbiont, PedF, KS8 [γ, Pseudomonadales] (AAS47564)
Labrenzia sp. PHM005, PKS/NRPS15, KS8 [α, Rhizobiales] (WP_141184176)
Entomycale ignis, MycF, KS8 [γ, -]
Entotheonella factor, OnnI, KS8 [Tectomicrobia] (AAV97877)
Endobugula sertula, BryC, KS6 [γ, Pseudomonadales] (ABK51301)
Psammocinia aff. bulbosa symbiont, PsyD, KS7 [?] (ADA82585)
Profftella_Dco, DipP, KS3 [γ, Burkholderiales]
Profftella_DC, DipP, KS3 [γ, Burkholderiales] (WP_020915398)
Paederus fuscipes symbiont, PedI, KS3 [γ, Pseudomonadales] (AAR19304)
Labrenzia sp. PHM005, PKS4, KS3 [α, Rhizobiales] (WP_141184165)
Nostoc sp. N6, NspA, KS3 [Cyanobacteria] (ADA69237)
Cuspidothrix issatschenkoi, CusA, KS3 [Cyanobacteria] (WP_133163982)
Entotheonella factor, OnnB, KS3 [Tectomicrobia] (AAV97870)
Entomycale ignis, MycI, KS3 [γ, -]
Psammocinia aff. bulbosa symbiont, PsyA, KS2 [?] (ADA82581)
Profftella_Dco, DipT, KS9 [γ, Burkholderiales]
Profftella_DC, DipT, KS9 [γ, Burkholderiales] (WP_020915462)
Paederus fuscipes symbiont, PedF, KS9 [γ, Pseudomonadales] (AAS47564)
Entomycale ignis, MycF, KS9 [γ, -]
Entotheonella factor, OnnI, KS9 [Tectomicrobia] (AAV97877)
Labrenzia sp. PHM005, PKS/NRPS15, KS9 [α, Rhizobiales] (WP_141184176)
Endobugula sertula, BryA, KS2 [γ, Pseudomonadales] (ABK51304)
Endobugula sertula, BryD, KS12 [γ, Pseudomonadales] (ABK51303)
Endobugula sertula, BryB, KS7 [γ, Pseudomonadales] (ABK51300)
Paraburkholderia rhizoxinica, RhiE_KS13 [γ, Burkholderiales] (CAL69893)
Bacillus velezensis, DifI, KS8 [Firmicutes] (WP_139138477)
Psammocinia aff. bulbosa symbiont, PsyD, KS9 [?] (ADA82585)
Burkholderia thailandensis, TaiD, KS2 [γ, Burkholderiales] (WP_146032765)
Chitinophaga pinensis, ElsP, KS12 [Bacteroidota] (WP_012792889)
Profftella_Dco, DipT, KS7 [γ, Burkholderiales]
Profftella_DC, DipT, KS7 [γ, Burkholderiales] (WP_020915462)
Paederus fuscipes symbiont, PedF, KS7 [γ, Pseudomonadales] (AAS47564)
Labrenzia sp. PHM005, PKS/NRPS15, KS7 [α, Rhizobiales] (WP_141184176)
Entomycale ignis, MycF, KS7 [γ, -]
Entotheonella factor, OnnI, KS7 [Tectomicrobia] (AAV97877)
Psammocinia aff. bulbosa symbiont, PsyD, KS6 [?] (ADA82585)
Paraburkholderia rhizoxinica, RhiD, KS9 [γ, Burkholderiales] (WP_013435480)
Paraburkholderia rhizoxinica, RhiC, KS7 [γ, Burkholderiales] (WP_013435481)
Psammocinia aff. bulbosa symbiont, PsyD, KS8 [?] (ADA82585)
Streptomyces albus, OzmH, KS6 [Actinobacteriota] (ABS90471)
Nostoc sp. N6, NspC, KS7 [Cyanobacteria] (ADA69239)
Cuspidothrix issatschenkoi, CusC, KS7 [Cyanobacteria] (WP_104385913)
Profftella_Dco, DipT, KS6 [γ, Burkholderiales]
Profftella_DC, DipT, KS6 [γ, Burkholderiales] (WP_020915462)
Entotheonella factor, OnnI, KS6 [Tectomicrobia] (AAV97877)
Paederus fuscipes symbiont, PedF, KS6 [γ, Pseudomonadales] (AAS47564)
Labrenzia sp. PHM005, PKS/NRPS15, KS6 [α, Rhizobiales] (WP_141184176)
Entomycale ignis, MycF, KS6 [γ, -]
Profftella_Dco, DipP, KS2 [γ, Burkholderiales]
Profftella_DC, DipP, KS2 [γ, Burkholderiales] (WP_020915398)
Paederus fuscipes symbiont, PedI, KS2 [γ, Pseudomonadales] (AAR19304)
Entomycale ignis, MycI, KS2 [γ, -]
Labrenzia sp. PHM005, PKS4, KS2 [α, Rhizobiales] (WP_141184165)
Entotheonella factor, OnnB, KS2 [Tectomicrobia] (AAV97870)
Nostoc sp. N6, NspA, KS2 [Cyanobacteria] (ADA69237)
Cuspidothrix issatschenkoi, CusA, KS2 [Cyanobacteria] (WP_133163982)
Psammocinia aff. bulbosa symbiont, PsyD, KS5 [?] (ADA82585)
Nostoc sp. N6, NspC, KS8 [Cyanobacteria] (ADA69239)
Cuspidothrix issatschenkoi, CusC, KS8 [Cyanobacteria] (WP_104385913)
Labrenzia sp. PHM005, PKS/NRPS13, KS13 [α, Rhizobiales] (WP_141184174)
Entomycale ignis, MycH, KS13 [γ, -]
Nostoc sp. N6, NspC, KS6 [Cyanobacteria] (ADA69239)
Cuspidothrix issatschenkoi, CusC, KS6 [Cyanobacteria] (WP_104385913)
Bacillus velezensis, MlnF, KS9 [Firmicutes] (WP_059367069)
Streptomyces virginiae, VirA, KS4 [Actinobacteriota] (WP_033220290)
Entomycale ignis, MycH, KS12 [γ, -]
Labrenzia sp. PHM005, PKS/NRPS13, KS12 [α, Rhizobiales] (WP_141184174)
Profftella_Dco, DipT, KS5 [γ, Burkholderiales]
Profftella_DC, DipT, KS5 [γ, Burkholderiales] (WP_020915462)
Paederus fuscipes symbiont, PedG, KS5 [γ, Pseudomonadales] (AAS47564)
Entomycale ignis, MycF, KS5 [γ, -]
Entotheonella factor, OnnI, KS5 [Tectomicrobia] (AAV97877)
Psammocinia aff. bulbosa symbiont, PsyD, KS4 [?] (ADA82585)
Bacillus velezensis, BaeJ, KS1 [Firmicutes] (WP_129192901)
Bacillus velezensis, BaeM, KS10 [Firmicutes] (WP_139138489)
Nostoc sp. N6, NspC, KS5 [Cyanobacteria] (ADA69239)
Cuspidothrix issatschenkoi, CusC, KS5 [Cyanobacteria] (WP_104385913)
Labrenzia sp. PHM005, PKS/NRPS15, KS5 [α, Rhizobiales] (WP_141184176)
Nostoc sp. N6, NspD, KS9 [Cyanobacteria] (ADA69241)
Profftella_Dco, DipP, KS1 [γ, Burkholderiales]
Profftella_DC, DipP, KS1 [γ, Burkholderiales] (WP_020915398)
Paederus fuscipes symbiont, PedF, KS1 [γ, Pseudomonadales] (AAR19304)
Nostoc sp. N6, NspA, KS1 [Cyanobacteria] (ADA69237)
Cuspidothrix issatschenkoi, CusA, KS1 [Cyanobacteria] (WP_133163982)
Entomycale ignis, MycI, KS1 [γ, -]
Chitinophaga pinensis, ElsI, KS1 [Bacteroidota] (MWH57224)
Psammocinia aff. bulbosa symbiont, Psy, KS1 [?] (ADA82581)
Entotheonella factor, OnnB, KS1 [Tectomicrobia] (AAV97870)
Labrenzia sp. PHM005, PKS4, KS1 [α, Rhizobiales] (WP_141184165)
Saccharopolyspora erythraea, DEBS3, KS5 [Actinobacteriota] (WP_009950919)
Saccharopolyspora erythraea, DEBS2, KS4 [Actinobacteriota] (WP_011873138)
Sorangium cellulosum, EpoD, KS3 [Myxococcota] (AAF26921)

Fig. S2.- Phylogenetic position of ketosynthase (KS) domains in DipO_Dco, DipP_Dco and DipT_Dco proteins inferred by the maximum likelihood method. A total of 507 aligned amino acid sites of 114 *trans*-AT PKS KS amino acid sequences from 20 *trans*-AT PKS biosynthetic gene clusters were subjected to the analysis. On each branch, bootstrap support values over 50 are shown. The scale bar indicates the substitutions per site. Source organisms, specific protein names (if applicable), and KS numbering based on the biosynthetic order, are indicated in this order. Higher bacterial taxa based on GTDB taxonomy (Parks et al. 2018) are shown in brackets. α and γ indicate classes of the Proteobacteria. Note that the former class Betaproteobacteria is reclassified as Burkholderiales, an order within the class Gammaproteobacteria. DDBJ/EMBL/GenBank accession numbers (if applicable) are provided in parenthesis. Three *cis*-AT PKS KS sequences from the *Sorangium cellulosum* epothilone and the *Saccharopolyspora erythraea* erythromycin pathway were used as an outgroup. Sequences from *Profftella* are highlighted in red. *Profftella*_Dco sequences from this study are shown in bold.
