# Supplementary material for: Comparative Genomics Underlines Multiple Roles of Profftella, an Obligate Symbiont of Psyllids: Providing Toxins, Vitamins, and Carotenoids
Source: Genome Biol Evol. 2020 Aug 14;12(11):1975–87. doi: 10.1093/gbe/evaa175 (PMC7643613; doi:10.1093/gbe/evaa175)
Supplement: evaa175_Supplementary_Data [file evaa175_supplementary_data.zip › FigS3_CrtI_tree_200613.pdf]

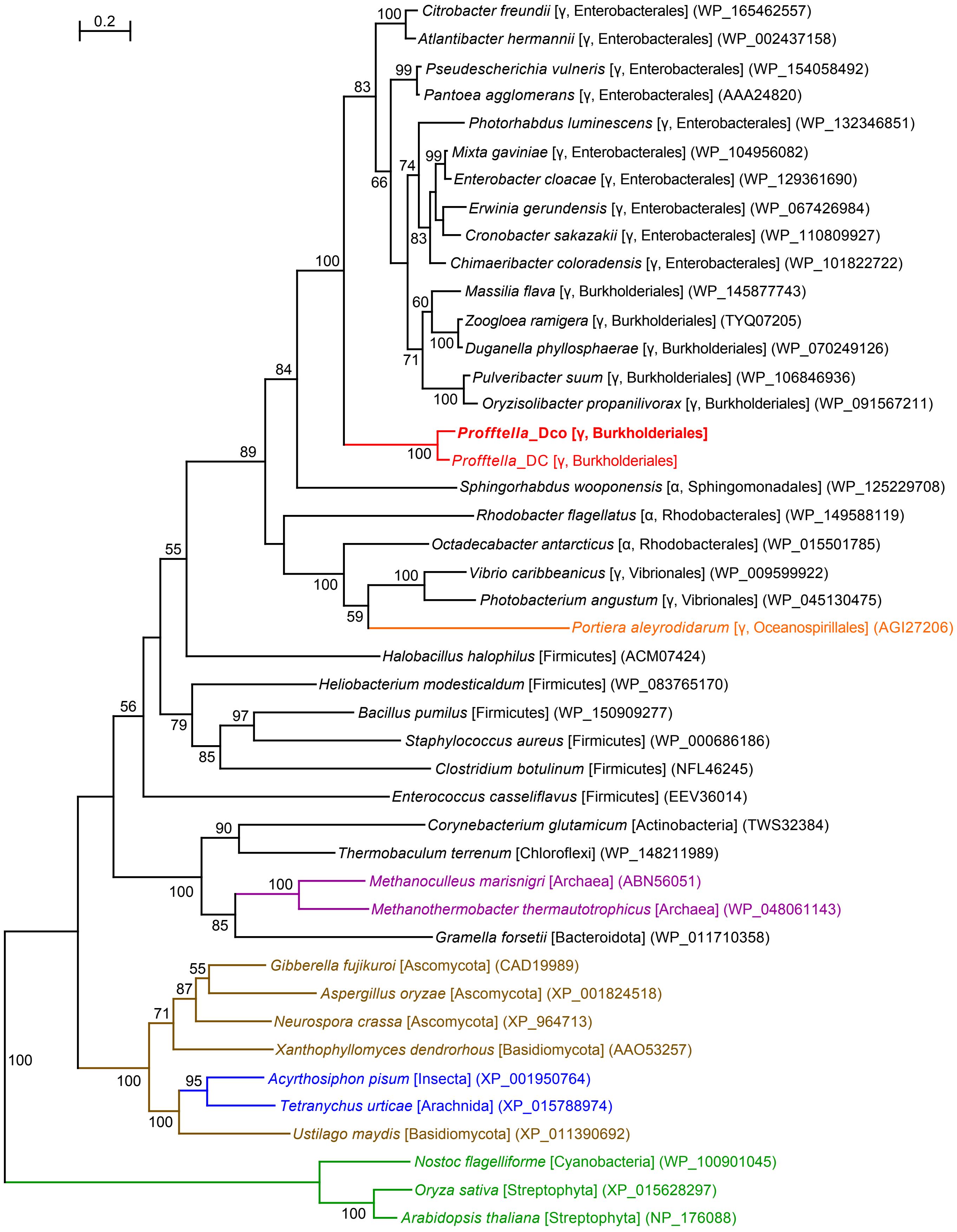
0.2
Citrobacter freundii [γ, Enterobacterales] (WP_165462557)
Atlantibacter hermannii [γ, Enterobacterales] (WP_002437158)
Pseudescherichia vulneris [γ, Enterobacterales] (WP_154058492)
Pantoea agglomerans [γ, Enterobacterales] (AAA24820)
Photorhabdus luminescens [γ, Enterobacterales] (WP_132346851)
Mixta gaviniae [γ, Enterobacterales] (WP_104956082)
Enterobacter cloacae [γ, Enterobacterales] (WP_129361690)
Erwinia gerundensis [γ, Enterobacterales] (WP_067426984)
Cronobacter sakazakii [γ, Enterobacterales] (WP_110809927)
Chimaeribacter coloradensis [γ, Enterobacterales] (WP_101822722)
Massilia flava [γ, Burkholderiales] (WP_145877743)
Zoogloea ramigera [γ, Burkholderiales] (TYQ07205)
Duganella phyllosphaerae [γ, Burkholderiales] (WP_070249126)
Pulveribacter suum [γ, Burkholderiales] (WP_106846936)
Oryzisolibacter propanilivorax [γ, Burkholderiales] (WP_091567211)
Profftella_Dco [γ, Burkholderiales]
Profftella_DC [γ, Burkholderiales]
Sphingorhabdus wooponensis [α, Sphingomonadales] (WP_125229708)
Rhodobacter flagellatus [α, Rhodobacterales] (WP_149588119)
Octadecabacter antarcticus [α, Rhodobacterales] (WP_015501785)
Vibrio caribbeanicus [γ, Vibrionales] (WP_009599922)
Photobacterium angustum [γ, Vibrionales] (WP_045130475)
Portiera aleyrodidarum [γ, Oceanospirillales] (AGI27206)
Halobacillus halophilus [Firmicutes] (ACM07424)
Heliobacterium modesticaldum [Firmicutes] (WP_083765170)
Bacillus pumilus [Firmicutes] (WP_150909277)
Staphylococcus aureus [Firmicutes] (WP_000686186)
Clostridium botulinum [Firmicutes] (NFL46245)
Enterococcus casseliflavus [Firmicutes] (EEV36014)
Corynebacterium glutamicum [Actinobacteria] (TWS32384)
Thermobaculum terrenum [Chloroflexi] (WP_148211989)
Methanoculleus marisnigri [Archaea] (ABN56051)
Methanothermobacter thermautotrophicus [Archaea] (WP_048061143)
Gramella forsetii [Bacteroidota] (WP_011710358)
Gibberella fujikuroi [Ascomycota] (CAD19989)
Aspergillus oryzae [Ascomycota] (XP_001824518)
Neurospora crassa [Ascomycota] (XP_964713)
Xanthophyllomyces dendrorhous [Basidiomycota] (AAO53257)
Acyrthosiphon pisum [Insecta] (XP_001950764)
Tetranychus urticae [Arachnida] (XP_015788974)
Ustilago maydis [Basidiomycota] (XP_011390692)
Nostoc flagelliforme [Cyanobacteria] (WP_100901045)
Oryza sativa [Streptophyta] (XP_015628297)
Arabidopsis thaliana [Streptophyta] (NP_176088)
100
99
83
74
66
99
83
100
60
100
71
100
100
84
89
55
100
100
59
56
79
97
85
90
100
100
85
55
87
71
100
95
100
100
100

Fig. S3.- Phylogenetic relationship of phytoene desaturases (CrtI) inferred by the maximum likelihood method. A total of 515 aligned amino acid sites of 44 CrtI orthologs were subjected to the analysis. On each branch, bootstrap support values over 50 are shown. The scale bar indicates the substitutions per site. Source organisms are indicated with higher taxa in brackets. Bacterial taxa are based on GTDB taxonomy (Parks et al. 2018). α and γ indicate classes of the Proteobacteria. Note that the former class Betaproteobacteria is reclassified as Burkholderiales, an order within the class Gammaproteobacteria. DDBJ/EMBL/GenBank accession numbers (if applicable) are provided in parenthesis. Sequences from *Profftella* are highlighted in red. The *Profftella*_Dco sequence from this study is shown in bold. The sequence from *Portiera* is shown in orange. Sequences from other bacteria are shown in black. Archaea, fungi, arthropods, and plants are shown in purple, brown, blue, and green, respectively.
