# Supplementary material for: Comparative Genomics Underlines Multiple Roles of Profftella, an Obligate Symbiont of Psyllids: Providing Toxins, Vitamins, and Carotenoids
Source: Genome Biol Evol. 2020 Aug 14;12(11):1975–87. doi: 10.1093/gbe/evaa175 (PMC7643613; doi:10.1093/gbe/evaa175)
Supplement: evaa175_Supplementary_Data [file evaa175_supplementary_data.zip › FigS4_Carsonella_genome_200613.pdf]

A

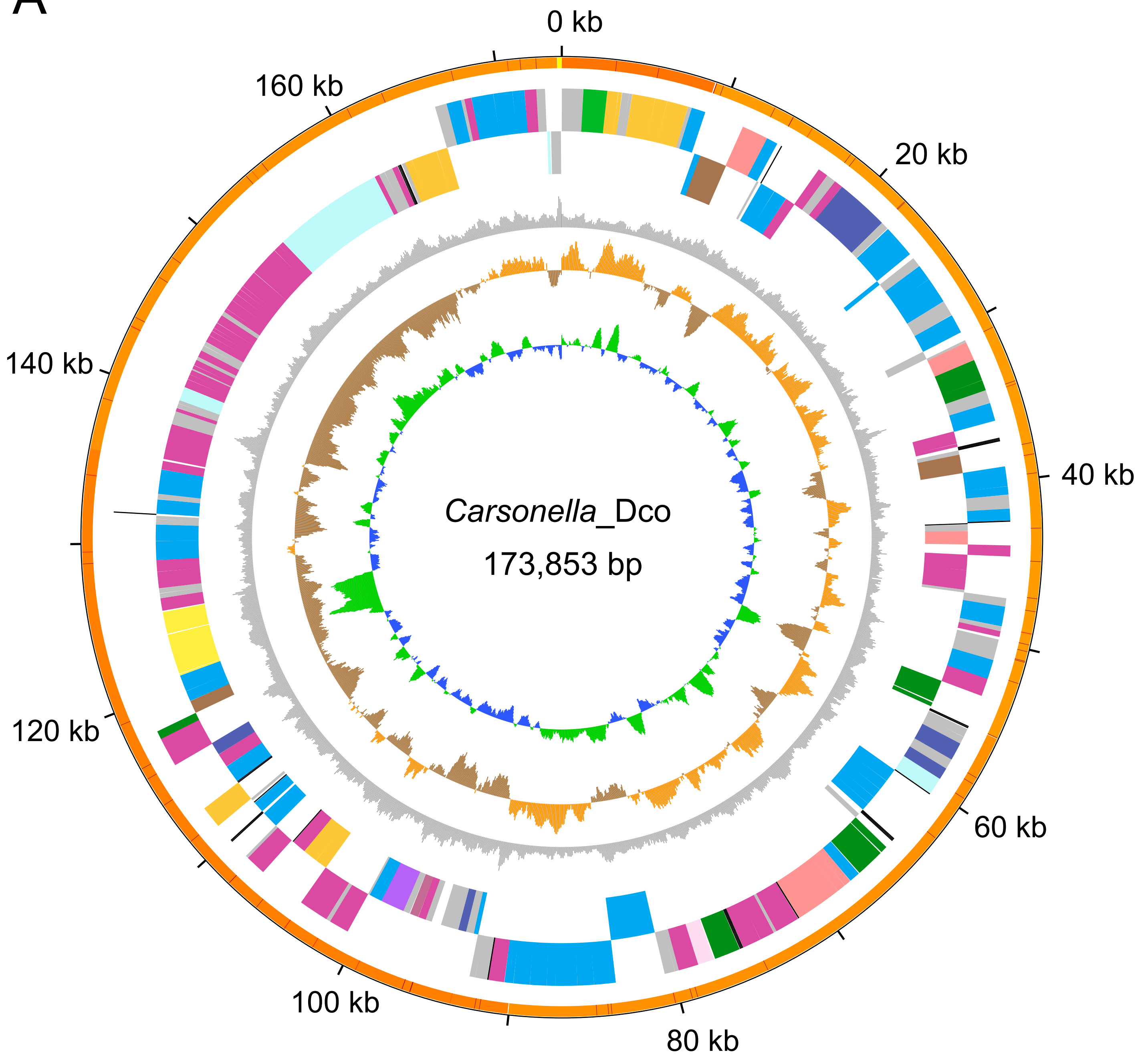

0 kb
20 kb
40 kb
60 kb
80 kb
100 kb
120 kb
140 kb
160 kb
*Carsonella*_Dco
173,853 bp

Color scale for (ii) sequence similarity to the corresponding symbiont genome in *D. citri*.

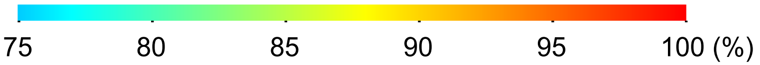

Functional categories of (iii, iv) genes encoded by *Profftella*_Dco and *Carsonella*_Dco.

- [Q] Secondary metabolites biosynthesis, transport and catabolism
- [L] DNA replication, recombination and repair
- [K] Transcription
- [J] Translation, ribosomal structure and biogenesis
- [O] Posttranslational modification, protein turnover, chaperones
- [C] Energy production and conversion
- [G] Carbohydrate transport and metabolism
- [I] Lipid transport and metabolism
- [F] Nucleotide transport and metabolism
- [E] Amino acid transport and metabolism
- [H] Coenzyme transport and metabolism
- [P] Inorganic ion transport and metabolism
- [M] Cell wall/membrane/envelope biogenesis
- [D] Cell cycle control, cell division, chromosome partitioning
- [N] Cell Motility
- [U] Intracellular trafficking, secretion, and vesicular transport
- [T] Signal transduction mechanisms
- [R] General function prediction only / [S] Function unknown / Not in COGs
- rRNA
- tRNA

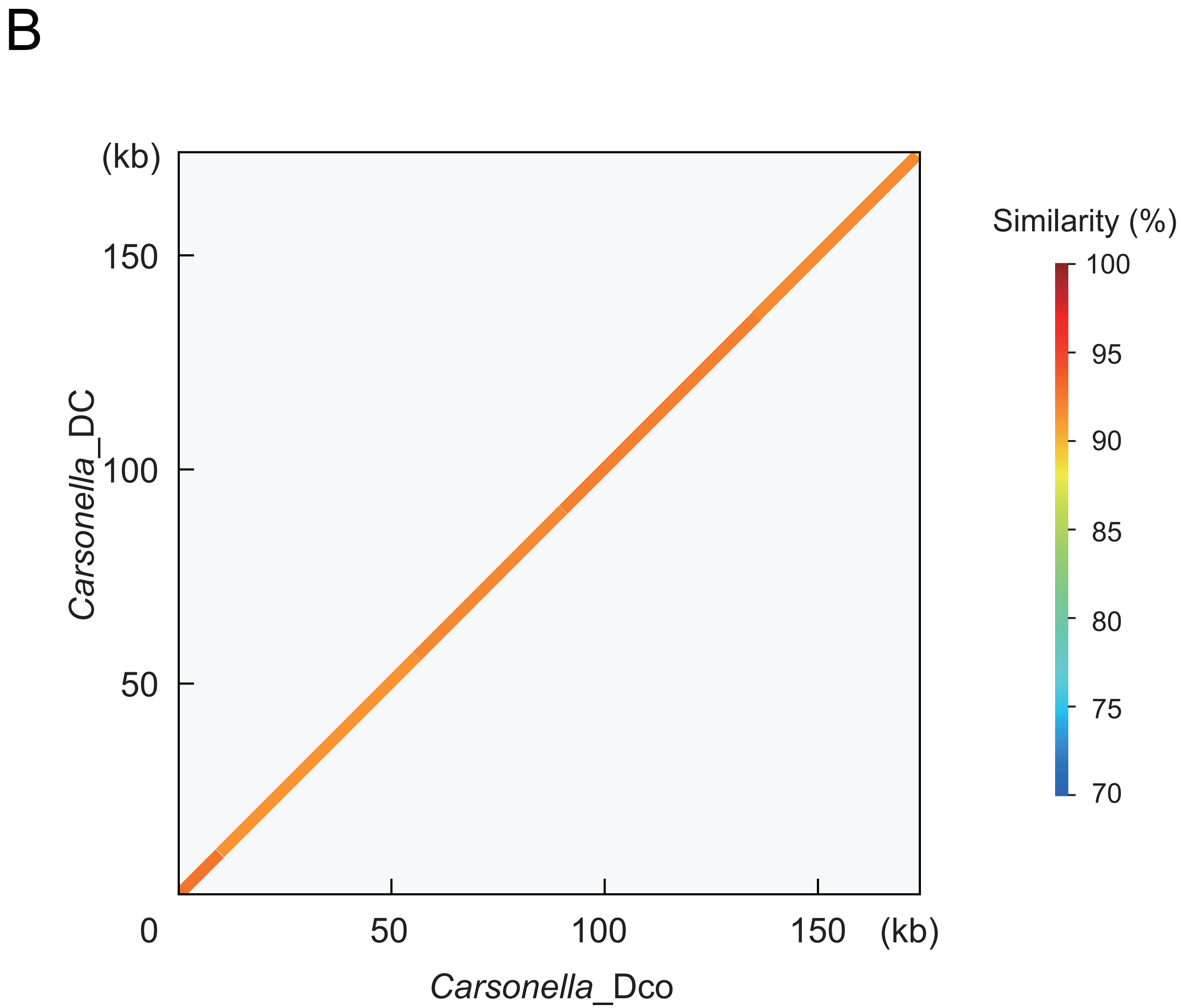
B
(kb)
150
100
50
0
50
100
150
(kb)
Carsonella_DC
Carsonella_Dco
Similarity (%)
100
95
90
85
80
75
70

Fig. S4.- Structure of the *Carsonella*_Dco genome. (A) Circular representation of the genome of *Carsonella*_Dco. The concentric rings denote the following features (from the outside): (i) the scale in kilobases, (ii) nucleotide similarity to the *Carsonella*_DC genome, (iii) forward strand genes, (iv) reverse strand genes, (v) dinucleotide bias, (vi) GC skew, and (vii) G + C content. For the calculation of (v), (vi), and (vii), sliding windows of 1,000 bp and a step size of 100 bp were used. (B) Comparison of the genomic structures of *Carsonella*_Dco and *Carsonella*_DC. The genomes of *Carsonella*_Dco and *Carsonella*_DC are represented by the x and y axes, respectively. The thick line indicates the shared synteny between the two genomes. The color of the line indicates the percentage similarity between the nucleotide sequences.
