# Supplementary material for: Comparative Genomics Underlines Multiple Roles of Profftella, an Obligate Symbiont of Psyllids: Providing Toxins, Vitamins, and Carotenoids
Source: Genome Biol Evol. 2020 Aug 14;12(11):1975–87. doi: 10.1093/gbe/evaa175 (PMC7643613; doi:10.1093/gbe/evaa175)
Supplement: evaa175_Supplementary_Data [file evaa175_supplementary_data.zip › Text_S1_Dips_200503.pdf]

Supplementary text S1.

Sequence of PKS proteins of the diaphorin pathway in *Profftella*_Dco. The domain architecture is shown at the beginning of each protein and corresponds to color-coded parts of the sequence. Abbreviations: A, nonribosomal peptide synthetase adenylation domain; ACP, acyl carrier protein; C, NRPS condensation domain; CR, crotonase (alternative name: enoyl-CoA hydratase); DH, dehydratase; KR, ketoreductase; KS, ketosynthase; KS0, non-elongating KS; MT, methyltransferase; PCP, NRPS peptidyl carrier protein; PS, pyran synthase; ?, unknown. Domains that are predicted to be nonfunctional (based on the absence of catalytically important residues) are shown in parentheses. In the ACP and PCP sequences, the conserved motif harboring the catalytically important serine residue is underlined.

DipP_Dco

GNAT ACP KS1 KR MT ACP KS2 ECH ECH ACP ACP ACP ACP ACP ACP KS3 ? KR ACP

MHKILENFFKKNENSLEKCFLEAAKIGLFITHQPPKNLLDIKSIKNYILFNLKYKPYCIRTALLE
DIPELIKLEKSCWKKLLRTPEIIIKNRICNYPKGQLVLLINNKISGVIYSQLIKSVKELENHNIT
TVDTLHNIHGSIAQLLAINIFPDMQQQNLGDQLLEFILIYYSLQTKINLVIAITLCKNFNKLDNL
SLSEYIKKRNIYGMLIDPILYFHESHGAIIKKLIPNYRPFDYKNNGYGVLINYDIKNRKCIDINN
KTKVKKNNKYNLEEISTYIKKIVYECLSFEFKKFFSIDTPLMDMGLDSSDLLFLNKNLSSHYQLN
IDSTFLFYNNTVNKIIFYFYNKINNIDVFKKNNKSYLLGKNFKNIDNNSLNNLNKDIAIIGLACH
LPGGINTLSKFWKILKKGSSVIEELHENRWIWPIEIDIKNKHKGINFGAFLKDIEYFDAPFFRIS
PIEAKSMDPQQRILLELSWKTIEDAGYNSNELSGSNTGVFIGASGSDYTRLLSDYIKIPINAHYC
TGNSMAVLANRISYFYNFTGPSLLIDTACSSSLVAVHAALQSIKNGESSQALVGGINLILHPFNT
IAYYKAGMLSRDGICKTFDKNADGYVRGEGAVMLMLKSLSVALKDNDNIYAIIKGSASTHNGQSN
GLTAPNPIQQSYLLQKAWNTSNIDVRSLGYIECHGTGTKLGDPIEIQGLKNAFHSIFSYEKNIKN
FYEKCGLGSVKTNLGHLEAAAGITSILKVILCLKNKKLPASLNFYNLNNNIKLDDSHLYVIDHFQ
DWVKPRYENRLRMAGISSFGSGGTNAHVVLSEYNIKINNKIINNISINSTNFTSPLIFVLSAKTK
LQLLIYVKKYIEWLNNKENLNISLRDLTLQLQKGRQAMSCRLALVIFNRDNLLFQLNFFYKKKII
PLQRELDLFNDLKNKKDMVAVINSAIKKKNFKKIAILWQSGIDIDWSLIDKKNTRISLPTYPFSK
YYYWLPKNNFNKYISNSDDTHISNSDDTHISNSDDTHISNSDDTHISNSDDTHISNSDDTHISNS
DDTHISNSDDTHISNSDDTHISNSDDTHISNSDDTHISNSDDTHISNSDVMQLSLTWDPIIMNYE
NNSYLINRKLLVIGANFKQKKIIQSINCSMYEFFINITESVDIISYKLNKLISEGCKFDHIIWIT
PQIFKIDFINYQDFGVLLLFKFIKALLSQKIYSSRELVWTIITYSTQEVFDTDLVSPGYASIHGL
AGVLAKEYPFWIINVLDLEFNSKLEDILVNIPKNIINGEVLAYRNGQWFKRYLYSIVNLRLSSKP
IYKQKGVYIVIGGSGGIGEAWTNYVIKKYSAQVIWICRSKLDVNIQKKLEKFKKINSSILYISAD
ACDLTSLKKSLTIIKKIYPVINGVIHSAVSSEFDQSIEKMSIKRFQSILSIKIDASINIAKVFST
EVSDFIFYFSSIVAIEKNGGMSGYSSGGAFEDAYAICLSKKLQCFVRVINWGHWEIGTGEKISNF
AKIRLKQSGRLLLKVSQAMTFLENISNIPLRQIIVIRVSCPKSLFLFNSLKNLIIYPDNLDIITP
FSLNFMLTESDKKVELLKNFSIFNNKIMEIKLLPLLAGILVSMGFLGKDKNNHWKILRNISNNTP
KFYEKWLKASIKFLKKYNKKNILIVLENLNNLEKLWEFWNISKQKYFNNSNLESAIILVETCLRS
LPLILSGKIRSTDILFPNASMRMVNGIYCNNLVSDYFNKILAKTIVSFIKLRLQKEPLARIRIIE
IGAGTGGTTSIILPYLNPFYKNISEYAYTDISKAFLFHAEKKFLSNYPYLTPKIFNIEKPLSNQM
IKPGSYDIAIATNVLHATRNICFTLSNVKAVLRKNGLLLLNEISKKSFFAHLTFGLLEGWWLSDD
QRIRISDSPGLCSKNWYKILSQEGFNTILFPAKNTHNLGQQIIISKSNGIIKQKISMEKKIENNQ
LKSIHKDKNFSLKVIILYLKKIIAKVLHMDISKIDSQELLGNYGIDSILIIQITNKLRKDFSNIS
STLLFEYKNIFELANYLIDNHKKELQELTKIKIDNKKIDNKKIDNKKIDNKKIDNKKIDNKKYEI
KELKINTLSKNTIDICDNNDIAVIGMSCHFPKANNLKEYWQLLINGKNCITEIPFSRWEIDKFYH
PDPEEAIKQGKTYCKWGGFLNKIMNFDPLFFNISPKEANIIDPQERLFLQTAWETLEDAGYTRER
LARDFNHQMGVFVGVTRTGFDLFGPELWKCGSRLYPHTSFSSFANRVSYFLNIRGPSMPIDTMCS
SSLTAIHQACQSLRSGESKLAIAGGVNLYLHPSSYILLSSSRMLSKDGICRSFGENANGFVPGEG
VGAVLLKPISKAIEDNDRIQAIIKATHINHGGKTNGYTVPNPNAQFELIKNTLKKANINARNISY
IEAHGTGTELGDPIEISSLVKAFRNDTEDNNFCAIGSVKSNIGHLEAAAGIAGFIKVILQMKNKR
LVPTLHAKIANPNINFSKTPFFLQQTLVNWIREKNLPRVAGISSFGAGGSNAHIIIEEFKKIKKS
FNFSCEKKYIIILSARNEESLKNYIKCLYKFTLQMSYEKLANLSYTLQIGREAMDIRIAFIVCSL
SELQKKLQKCLNIQGNNYSFLEEEENIFYGKIKNYKDVISIFSNDITIQDTLKIWFHKKKYERLL
KFWSKGMNIDWTKLYSEKNMYHCIALPTYPFLETPYLLPLLKTKSDFSISSSNQVIDIKYNRLKY
INLKDHGKGIFSIKFSNFYNVNENIEEKIKNSCCELISCIKSLSNLDKVKVLILNNLDKLFADTI
NINKLIQTEISNIIQSLIKFLFPIIIVLTGKNSNHVMKISMTCDLIICSKQGYYCDLSSGIIFDS

KSLRKFKLSYPIIDESIIQNYAFEKANQIIRSSSISLKILKKHLNKNIDNIITNLRNFSQYPDFL
LKLSYNKDKISVIPFSSYVIKLEEYYNDILILTICDRENKNMFSSSVVKGIIDAFSYINSSLKYK
VIIITGYKNYFACGGTKNGLLKIQKGISRFTDEKSYCMPLYCKIPVIAAMQGHAIGAGWAMGLFC
DYTVFSKESVYQSPYMCYGFTPGAGSTLIFPQRFGKILSREILLTAGEFKGKELKERGISMPVLP
RKQVLFYSLKLAKKLSLLSRKELELQKKYYSYSLQKALSTVFFHELTMHDYTFVNNPRVITNIER
YFDKSIKKNDSKIISDTISYGDSKKLYSSSSIFSILKNSLIEELQIKPEQFDDDAVFIDMGLDSI
IAVTWIRKINSQLDLSINSTKIYDYPTLNKFFKFIMSQISKKLQLKNLSIDNNIDFDKSIKKNDS
KIISDTISYGDSKKLYSSSSIFSILKNSLIEELQIKPEQFDDDAVFIDMGLDSIIAVTWIRKINS
QLDLSINSTKIYDYPTLNKFFKFIMSQISKKLQLKNLSIDNNIDFDKSIKKNDSKIISDTISYGD
SKKLYSSSSIFSILKNSLIEELQIKPEQFDDDAVFIDMGLDSIIAVTWIRKINSQLDLSINSTKI
YDYPTLNKFFKFIMSQISKKLQLKNLSIDNNIDFDKSIKKNDSKIISDTISYGDSKKLYSSSSIF
SILKNSLIEELQIKPEQFDDDAVFIDMGLDSIIAVTWIRKINSQLDLSINSTKIYDYPTLNKFFK
FIMSQISKKLQLKNLSIDNNIDFDKSIKKNDSKIISDTISYGDSKKLYSSSSIFSILKNSLIEEL
QIKPEQFDDDAVFIDMGLDSIIAVTWIRKINSQLDLSINSTKIYDYPTLNKFFKFIMSQISKKLQ
LKNLSIDNNIDFDKSIKKNDSKIISDTISYGDSKKLYSSSSIFSILKNSLIEELQIKPEQFDDDA
VFIDMGLDSIIAVTWIRKINSQLDLSINSTKIYDYPTLNKFFKFIMSQISKKLQLKNLSIDNNID
FDKSIKKKNKKQKNIFLNIFDKNNKKFNNLVSYDIAIIGISGQFPKANNVKKFWNNLLIGRDCVS
KIPNIRWFIDDFYDSDKNAIGKTICKKMGSLEDVEIFDPLFFNISPSEAEYMDPQQRLFLQNCWT
CIEDAGYNPFDLSGSLCGVFVGCAVSDYNKLITDPDEIHNAYALMGESVSILPARISYFLNLQGP
CLAIDTACSSSLVAIANACDSLVLGNSKIALAGGVYIINTPDIHIKMSKSGMLSPDGHCYSFDQR
ANGFVPGEGVGVLMLKKLEDAKKDGDDIYSIIKGWGVNQDGKTNGITAPNTQSQVRLQTEIYKKF
DINPDDIQLIEAHGTGTKLGDPIEFEALCESFNKFSYRKNFCALGSVKSNIGHSATAAGVIGIIK
IILSLKHKILPPTINYRILNEHIKLENSPFFINTQCKSWNIENKKKRMAAINSFGFSGTNSHIVL
SEKIKDNKNIKFINNLPIFTVFPLSAKNLSQLLVYSKLICNYISKDLKNNNINIFDFIYTFQFGR
AVMSYRLAVVISSFEELYEKLLNFIKNYKDKENYIDNNFFYGKIKSKKNNTIFKKLKNLEMTNKN
SLEISIILANSWVNGDAVDWKYLQLFNKNLGYRKHGLPTYPFAREYYWIFKEQKKNIFHKKNNFK
LSNINYKSNIYNELLDSIDLQDRLQFYKNKQIIFIYTDKNEFNIFKKLLSQLQKVSNLEDKKTNN
FISPIYYNFYSFFTKKLFFEEKYEIVLIFNISKISDDIIILLEKFFQKFLNGYFGYPIEIIFFIQ
LEISNKEYINKFINYKIHLSINNLSKLKRKKIIFCDDYNKFNRLFFIQRLCIEWLIPEKNNTLVD
FNYIYYLNNKRFIKENLINKNSEENNIYLISKDWRVKEPYFLKNNIERKILLVLVNEDSIQLVEK
ILNLNDFKKIILISIFNSGINYISKYDLVNLNDINSARINIQSLVNKYDNNITHIIDLSDIYNVT
HDYDNDQFEKIVFYQIIINTINEISILYFTKELQNFKFKKMTLSGSKFSGIIKMISSDYQHINSR
CIDIDNSIYNEPLKLRNIIFKEFNIKLQETEICYRDNQRYVPILLEKKLEIKNYMPFLISNKGVY
VITGGTNGVGLEIAKYFVEKGCKNIVLMGITNLPPKEDWKKIISDNDLSFYLINKLKELIYLERK
IKNLKIYIGSLDDLNSLEKYFIKIRNNYGPIKGVIHCAGVYSNIKKPGFSNKNIEDIKKVWEPKI
KGIENLHKIFKLDPLDFFISFSSMSSLIPYLARGSSDYAAANTYMSFFSTYQQFLYKKIFYKNII
ISDWNQTGAITRISKEKFIIVEKVFNKLGMRTFNNKEGKILFEKIMNLNNENSIKENNNSIIINY
LNLSIFNNIKERLLYARPFKFKSKLMKKNIEDYIKYWEEKKKNGIKISIQEIIKIIDINEIKKLS
SDFIDRIHKLITFNKKQKLEKFKENKNFNKQILEKIISKTIMEVLKLKEIDFTETFQSYGLDSIS
AMVLSTKLEKNLKYTIQPQWLIDFNSVKKLSVYLSTLIINNK

DipT_Dco

KS^{0}4 ACP C A PCP KS5 KR ACP KS6 KR MT ACP ACP ACP ACP ACP ACP ACP KS7
DH PS KR ACP ACP KS8 KR ACP KS9 (DH)

MKTFQKLRDIETAILQATSHSITNTKENITSLSIPEPIAIIGLSGMFPKSKSVDVFWKSLDSDIS
LIEEIPESRFNWRHVYDKKFMKENYSKWGGFIPNIDEFDAHFFNIPPGEAIIMDPRQRLLLMSVY
QTLIDAGYNPKSLKKSNTGVFIAIQENEYLQILKDANIDINEWYSQNCLLANKISYYFDFRGISD
IIDAQCPGSAVAIHRAVNSLRNKEIEQAIVGSVNILLRPEPFILLSSVKQLSSTNTVNSFGPYAD
GHIRAEGVSSVLLKPLSRAIQDGDLIYALIKNSSVNYNGQGGGSISAPNAESHINLIQDCYQKVN
IDPRDINYIEAQGMGNVLADLTEWKSFNCALKNIAKQNKIILEDKTCYISTIKPMTGHMESASGL
GALFKVIRSMHTKLIHKIIGFKDYHPDMDRENQPCLIANKTIYWLKNKNNTRLAGIHCYGMGGTN
SHLLIEEYKREILDIDKDIVPVLIIISAKTKSSLIIMTKKLYNFLAKKNKKYRLSDIAFTLQVGR
EAMKYRISWVIESLQELLVALKKFFESKKFNILDYMIPVYYGVLEIPCTLQKKLLNIENFQKKKY
TKTDLCEIASLWIDGKFYSWHLLYTNKKLYRIRLPGYPFEKQRYWINNHCKNLYNQEKISQNIKS
ATFLIKNKKDNILEIKEYIIKYISDMLQKPQSTIDINQHLYNFGIDSIFTVRLLRNISQKFNIET
KGRDLLKNPTINKLSKYFGEILNSPSILIKSEKEKKNKHLKEYIDYKYSKKQFPLSENQKGLWVL
QEIAPNMSAYNIPICFHITREINSDILQLAFLCTLQKYPILTSIFFQKNKKIIRENKLDKNIIIQ
KRDVSFIKDKKKIFEYIKTIIKKPFKLDKGPLVRLYLLKGINDYYLLFNIHHIIFDGKSFLPTIS
FFIKTYSKLLDANKDDTKLIVLNSKINSDDIYEKFVSWESDMLNGVEGKKYRNYWLKQLKNISSI
ELFTDYPRSTARLFEGEVYSCKLNSSLSYEIKNYAKKQNFNLSSFFLGAFNILISQYTGKNEIII
GMVEMGRSQEIFENSVGYFINMLPIRINNLMYSKESLNKFINRVQFIIADAIDNSAYPFSLMVRD
LRIKSNINFSPIFQIAFEYQNAFSKNDLLEFNKRFRKNFSITMIEEIMQLGEYELVLEIREEIDN
FILNFKYNPTLFKAFTITRMSEHIVHIIKEIIKNSNLIISEFSILLDKEYKLLEKWNKTIVDYPK
ELCFHHFFEKQAHISPNSIAVIFKEKKLTYFELNEKSNQLAYYLYDIWKKDLFQKNKLVAVCVNR
SLNMTIIFLGISKSGACWLPIDPLYPDERLCFMLNDSKVDMILTEKILLKRLQNLSNRKKINNSI
AIIELDNIQNNLLRNKSTLLPKVNSKSLAYIIYTSGSTGTPKGVMVKHRSLTNFLLSMANKPGFS
SEDRLLSVTTYCFDIAYLELFLPLITGGCCCICPSEKLNDAIELKKEIKRLQPTIMQATPSTWTM
LFSWKWKNTENLKILCGGESLPLFLNNKFLETDSEAWNMFGPTETTIWSIIACINTINKNNDFLK
KSNNIFIGKPIANTQVLVLDSIGRLSPIGIPGELCIGGDGLAKGYLNNLSLTSQKFITFSFRDKI
RVYKTGDLVRWSENGMLEYLGRLDQQVKILGYRIELGEIENILNKHTKIKKSVVIARNQQNNKQL
IAYYTKKYTEQIPPEELKKYLQKYLPFYMIPAIFINIADIPLLANGKINRNSLITREIIINQDNS
LELKKNIKTNKIINNKKNDNILKSKIQYKLLNIWCDLLNIKGIKVYDGFFSIGGNSVLSVILAQK
ISKDFNIKFYASDLFKYSTIEDISLYICDYFQQNNESKIINSKISTLKKNQSKIRKIVSDDKIVN
SFDDYEDCLAIIGISCNVPGAKNYRKFWKNLCAGKESSTKLSLDVLRKFGVPNNILNDKNFIPIQ
YNIEGKDLFDPEFFNISSKNAMFMDPQFRKLLEHSWGAVEDSGYISNQIPETAVFMSVSNNFYKT
LLYNSEMVDNKDEYSSWILNQSGTISTMISYQLGFKGPSVSIHTNCSSTLSGLYLAKQSLQLNEV
KYALIGGASLFPIPNIGHTYIPDMNFSSDGHCKAFDFSADGLVGGEGVAVIMVRKAIDAIKSGDH
IYAIVRGICINNDGSEKSGFYTPGIKGQTEVITKVLNKTGINPLSISYIEAHGTGTKLGDPAEII
ALNDAYKKYTNKKQFCGIGSVKSNIGHLDTVSGLIGLIKLALSLKYHKIPPSINYNKPNPEIKFE
STAFYVVDRLIKWPGNNIPRAGISSFGIGGTNAHAILEQYKIALENNNFTNFKNTQIIILSAKTN
IQLLKIAKNLKNYLEKEIIIDSQYSPQINLENIAFTLQTGRKAMNWRVAFVVNSIEILLNKLKKF
ISGKNIFENYFYGKIKFEKPIVINNNINNYQDCEYFNKIASDWVSGSLFDWLELHKNKKLNRISL
PTYPFKKEHYWVEIPKNKKKEFNSRDYNFLYKPYFYINKNKIIDYKKVIISYPKWCVQNITSLVK
NSSNLYFNKYLVFICGLNIENFNKIKSQMIDSQCISLQLNSNDTLIQQYTDVCLKIFNYIKNIIS
DNINLILIQLILVDRHAQSIFYGVGGLLKTAQLENPNIISQIIDVGDKNTSKFIIKKIIENRYFL
KDFFIRYRKNREKIIWKIFNDYDKDNLILPWKEGGIYLFSGGAGNLALLFSEEIINKLRSVTLIL
VYRSKLTLEQEMRITNLRHNKNISIILYKYDVNFLQNVNLVVQDIIKKFKKLDGVIHFSGSIHDN
FILKKNTEEFRTVLYNKIAGAFNLDYATRNLNIDFFILFSSIASIIGSVGQADYCAANGFLDAFA

HERNIRVKLGKAKGKTLSLNWPLWKDGGMRINKINENIVEENFGIKAISKNFGLISFYHSLLSKH
AQILILEGDKNILEKLLTFKKPNIKDIKKIPEKIFNNHNYDLKQKIIFQLKILLSKILKTPVEKI
QSTELMEKYGVDSIAITQINHELEKIFGNISKTLFYQYQTIDALSDYLINSYSDICSIWGKKFEI
CSDFLEKSLDINPNKESNNIKNFPEKQKSILANNSFFIPLHEPLCIIGISGCYPKSDTLDVLWDN
IKNGKNCITEIPKDRWSINNFFEKDRKKAVQKGRSYSKWGGFLNGFADFDPLFFNISPKEALGID
PQERLFLQCAWQVLEDAGLTRETLCKLHFKSLKEKEKFFNQIPRVGVFVGVTKTGFDLYGPEIRK
KRKMVFPHTSFSSIANRVSYFLNLSGPSMPIDTMCSSSLTAIHEACQHIRQGDCDIAIAGGVNLY
LHPSTYINLCSSYMLSNDGKCRSFGKGGNGFVPGEGVGAVLIKRLSKAIIDRDLIHAIIRGSSIN
HGGKTNGYTVPNPNAQTELIYMNIKKSGINARAISYVEAHGTGTELGDPIEITGLTEAFRRYTMD
IKFCGLGSIKSNLGHLEAAAGIAGLSKILLQMRYKKLAPTLHAREINPNINLETSPFFIQQKLEN
WPRPVIKINNKNKEYPRIASLSSFGAGGSNAHLLVEEYISDLNKINIEIRNTINFPIIILLTAKT
KECLKKVVINFLMWLNKKYNLFDNNFLKDIAYTLQVGREHMEERLGIIIKSIEELRNKLNYFLKE
NVYNSNLGIYRDQINIDKDSFSMLSDDTEFKETIKKWIILRKFNKLLPFWVKGLKIDWEILYLNE
TYKPKRIQLPLYPFKKEKYWIDSIIPKNKKNENFQKNNSQINNNKLIKLLPVWDILNTKELINDF
KINKLKKIIILGGNIEQKKEISKYYSRALELNSILKEDIFTISNKFNNINKFDHIVWIVPIYDIN
STYKNNVIIQLFRLIKALLREGYANINLQITIITVQGLLICDYDEINPDHASIHSLISSIAQEYP
IWNIKAIDMINNNVINWSVSLWYQLLEIKNISIARRKYNWFFQKLIPLNFYISKDIYCKNGVYLV
IGGAGGLGGIWSEFMIKNYEAKIIWIGRSPKNNILQEKIDNISIKYNGICPIYIQADARNSDLLC
SIYHKIKKDFDHINGIVVSTLSDYDLSFKEMSEDLFINILSTKRDVSLSIEKCFYKERLDFIVYF
SSMISFEKPGGMSAYVSSCVFSDAFAKKLNKKWNCAVKTINWGYWNTGGGIRISSALKKLIEKRG
VESINKEEGMNTLKFFLGNVKEFQQSAITCTNQPKFINTYSNKEFAIVLPNNISNIEKNILNDFH
SLENLKAPLKSKYTEELNYWIIRLLFVQLQLLGIFKVNCNYFKKNNAVDVRNKAGILDKYMVWWK
EVLNILHEYNYLITYKDIILVNKNISNIEVSYTETWRSWKKRKTYFLEKPETHTLAILVNDCLKE
LPKILHGDILVTDILFPNGSMEKIEGLYKNNCICDYFNDVVSSIVNSYIKNKIINNSMEGIKIIE
IGAGTGGTTSTILKKLEPWNNYINEYCYTDISKSFLNHACKFYGSKYPYLVYKLWNIEKSLISQD
INIGDYDIVIATNVLHATKNIRNTIRNVKAALKINGILILNEISNKTIFASVLFGLIDGWSLAED
TYLRIPGSPGLYSQDWFLLLQQEGYHNISFPVIREHKLGQQIIVAYSNGIIRQDSSLSTQNNSYK
LKSNLQNENFIAEKRIDKSVLINFIQNSILNALSESLMIQINIIKLNIPFSDYGIDSILGVNFIQ
KINNYLKLKLNTTILFDYTTVDSLTQYIITNYKEIIEKQYLSTVSKEKFVKPIINTNQNNVNKDH
IHYSSLSTQNNSYKLKSNLQNENFIAEKRIDKSVLINFIQNSILNALSESLMIQINIIKLNIPFS
DYGIDSILGVNFIQKINNYLKLKLNTTILFDYTTVDSLTQYIITNYKEIIEKQYLSTVSKEKFVK
PIINTNQNNVNKDHIHYSSLSTQNNSYKLKSNLQNENFIAEKRIDKSVLINFIQNSILNALSESL
MIQINIIKLNIPFSDYGIDSILGVNFIQKINNYLKLKLNTTILFDYTTVDSLTQYIITNYKEIIE
KQYLSTVSKEKFVKPIINTNQNNVNKDHIHYSSLSTQNNSYKLKSNLQNENFIAEKRIDKSVLIN
FIQNSILNALSESLMIQINIIKLNIPFSDYGIDSILGVNFIQKINNYLKLKLNTTILFDYTTVDS
LTQYIITNYKEIIEKQYLSTVSKEKFVKPIINTNQNNVNKDHIHYSSLSTQNNSYKLKSNLQNEN
FIAEKRIDKSVLINFIQNSILNALSESLMIQINIIKLNIPFSDYGIDSILGVNFIQKINNYLKLK
LNTTILFDYTTVDSLTQYIITNYKEIIEKQYLSTVSKEKFVKPIINTNQNNVNKDHIHYSSLSTQ
NNSYKLKSNLQNENFIAEKRIDKSVLINFIQNSILNALSESLMIQINIIKLNIPFSDYGIDSILG
VNFIQKINNYLKLKLNTTILFDYTTVDSLTQYIITNYKEIIEKQYLSTVSKEKFVKPIINTNQNN
VNKDHIHYSSLSTQNNSYKLKSNLQNENFIAEKRIDKSVLINFIQNSILNALSESLMIQINIIKL
NIPFSDYGIDSILGVNFIQKINNYLKLKLNTTILFDYTTVDSLTQYIITNYKEIIEKQYLSTVSK
EKFVKPIINTNQNNVNKDHIFYKTNFSSIPIIKNFSFSELNLDGIAIIGLSGKFPGAENVNVFWE
NMISGINPIIELPSHYLDSKYYNIKPQKGKTYCKWGGILSSRDCFDPMFFNISPRDAKSMNPHQR
LILQEGWRALEDAGYNPKILSESLTGIFIGAEPTSYIYKTFTGASDAIVASRLSYFLNLRGPSFV
VNTGCSSSAVAIHLACQSLRNGETNLALAGGVFAVMNQTILIGLAQTHMLSYSGLCNTFDSSADG
MVLSEGVGIVVLKLLKNAIIDKDPIYGIIRASGINQDGASNGITAPSGIAQQQLITSIYKRYKIN
PERITYIEAHGTGTKLGDPVEANALVNAFKKFTTKTNYCAVGSAKSYIGHTSANAGVTGLISVLL

SLKHHQLPGLRNFKKLNSLIQFKNSPFYPNIKTSLWNSIDNKPLMAAVNSFGHSGTNAHLVVEEF
IFLKKNITNFYKNNSSQLIILSAKDKTRLLEYIKKLLEFIIKILSKNNFFENSENLLSNIAYTLQ
VGRDSMESRIAFIVNDLNHFIKILKKIIKNNFNINNEFSENNIWVNDFELVNKIIIKDNIVKEWL
FFGNLKKLANAWIQGIVIDWDKLYRKNKNCNRIHLPTYPFEKERYWKPNNIKTEKKISPIFKKNI
LHPLVHENISTLNKQCFISTFHGHEFFLSDHVINNSKILPGVTYLEMAYFSVNYSINNYSQDKKS
FKLKNIIWIRPAFIHNKLLNISINLTKKSDNLINYSIYSDYDISLNKGKLLYNQGLAYIVSSKLE
SIYLNLSNLKNIFKNFEVEVDKCYMMLEKMNIFYGPSHKSLKIIYCTRNQVLAYLCLPDLIKDTC
DSYTLHPSLIDGAFQASIALKLNNIDFDNKKTLLPFSLEELEVFGKCEKEMWVWIRHNDNKSLEK
NDNKQRLNIDLCTNNGEIRIRIIGFTSRISINNNIEIISSKKNNSISHPLFIKNNFDQTKWTSHF
HGNEFFLKDHKNIMPAVIYLEMAYIAASKNTKITGLSNIIWPKILFIKKNNILKTQLIFKNINSI
KKYIIFDSQSKDVYCQGNFITNNCDSDQILKNTQVTDFCISDIKLLKSRCYSVLNYQQCNYILQY
THGPSLMSIYKLYSNNKEALALLNFPEKFLNKDNNYVFHPSLLNGAILSGVIWSIINRDKSKLIQ
NLPMPFSLKFFKIHDTSSFSEKLYIYIKKSKNFTLSKNLEFLNIQIFNTKGKLLISLLDLTLIFK
QPKYNLLYAIPKWKIEKLSSKIKIINNISKPIFLLLEKNIKLQNALYKAWPNSIINVIFNKNIQD
IEKNIFFEKFLIYIFKYCKNILKENNSIIQSLILLIPLEKKAFLYGGSISGFLKTACFEYTKLAA
KIIYYPVIKIDDFNQKLSNLIFNISTEISSISTDVEIYFSKEGKRQVQKLCEIDIKNNHENILFN
IKENDVIWVIGGMGGIGRLLTHYLGIIKKSQLILSGRSELTDKNLKFIEELSSKNINISYLKTNI
IEIESINTTLSIIKRKYGKLNGIIHCAGIIKDNYIINKTTSQFMRVVRPKISGLLNIDTATSNLP
INYIILFSSIAGFFGNIGQSDYATGNSFMDAFADYRNQCMYSGKRSGKTLSLNWPLWLEGGMKMN
VVNEKLMKKATGMTAMDNISGLIAFENVFSKNYNQILIAFGDYLEIKKRLLSFKKDSTYVNFKNH
EKEIVKFNSNKNLQLIQNEKKLSEMVRSELINIVAKVQHIPREKISFQKNLSAYGFDSISFTEFA
NVLNKTYELVLMPTLFFEIPTLIDLESYLFTHHKSELIKKHKSNYIKDEKKLSEMVRSELINIVA
KVQHIPREKISFQKNLSAYGFDSISFTEFANVLNETYELVLMPTLFFEIPTLIDLESYLFTHHKS
ELIKKHKSNYIKDEKKILNLKDSLENININTVAIIGIGGKFPGSKDINDFWRKLENNEDAITEVP
TSRWDWKAIYGDPHLESGKTKIKWGGFLSDADCFDAKFFGISPAEAEVMDPQLRVFIETTWATLE
DAGYPPSKLSGSKTAIFAGVSTADYKDILNEARRKGSVKSLAEPFPFMIANRVSYLFNFHGPSEV
IDTACSSSLIAINRAIESLHLKNCDLALAGGVNILASPNITIASSKAGLLSKNGRCMTFDQRANG
YVRSEGVGVLLLKPLKNAIIDNDHIYGIFRGNFENHGGHSSSPTSPNMLAQKQLLIDVYRRANIN
PCTISYIEAHGTGTKLGDPIEVNGLKSAFSELHKYYKTPLLKPYCGLGSVKANIGHLEAASGVIG
VIKVLLMLKYKKIPGNPHLKIPNSYLKLDNTPFYLVNKTCDWLQLDNNIPRRAGISSFGVGGSNV
HVIIEEYVQNIKINDIKENNILIPIIILSAKTKESLKKYVMLLLEFIIKEKNNFSLYDLAYTLQV
GREAMNYRLAIHVNSCEDLIKKLQDYLNKKITHGIYINYSEKDKNKNLEKINKTKNISILIDSWV
KKNRYDKLILSWINGSEFSWDKLYNIFKPKRINLPTYPFDRQKYWVSIKQKLVNQYNNIPIINEE
EKIYDKKSIFTFEEIWVKSPLISLPLFRVKKKNLLCFSSNKDFQSKIIKSISSYTNIIFIEQNDN
IKDIQGSLDHTVNYQINKNNINDYISVLNKIYEKDKKIDFVFYLWPLEDENCIYNIMPVIYFLQA
VKKSKLYLHKLIISGISINKKNEENLYYCYFNSWISFERSLYIIMPETQISIVFAEKITNFNIQS
WITILWKELQEPKLFSIMYYGNDLVRYTLNIKSIILKDFKNSYIKQGGTYLITGGTGRLGMLFSN
YLINKYSTKLILSGRSKLDSIIYNKLKKFNGKAIYIQVDASNKFKMISEINSIINNIGPIDGVLH
LAGISGLTSNILEANYKNFYSVLNSKISGTIALNYALLNTICRLQNNKLDFVCYFSSSSAILGDF
GSCDYAMGNRFQTAYAKYLNNKNIYKKYTNKLIVINWPLWNNSKFKIGNHEQADFYLKSSKQKIL
YENDGLKLFEQLLIQDKTQYLVLVGKLEKLNQIINNISPKISENKKINNISPKISENKKINNISP
KISENKKINNINIEKFIENDLKNHICNILNTKNNEIYKNKNLADYGFDSISLAEFSRILSKFYSL
DIMPSIFFSYSTLERLIIYFIKNHNDTMIEFYRNKYSFKIVDKKNISTLYTDKINKNFENISKKS
NFILNKNTDIDSEDLIAIIGMSGRFPAARNINDFWKILINNKDVIEEIPRKIFDWRLYYENPIKS
SNKINSKWYGAIPGIDEFDPLFFEISPLEAERMDPRQRHLLQESWLALEDAGYGPNQIENQKIGM
FVGVEEGSNYQDRLDQVNLTSTHNAILSARLAYFLDLKGPVMAINTACSSSLVATHVACQSLRQY
ECDTAISAGVNLMISPEAYIAMTNAGMLSPNGKCYVFDERANGLVPGEAVVAIVLKRLSRALFDG
DPIHAIIRGSGINYDGKTNGITAPNGISQTELIKSVYKKSNINPEDINYIVTHGTGTKLGDPVEI

NALYDVFKNKTCNKNFCAITSNKSNIGHTFAASGLVSLINLVQSIKYKIIPASLHCEKENNYIIW
KNSPFYINKSNKKWDVVNEKLRIGAVSAFGMSGTNAHIVLQEYISTNFKNIKNNIFISSNPYHIV
VLSAKTKISLKEKMKKILLFLKKNNTICIENFVYTLMQGRYHFQYRCAIIISSIKEIIKIINDIL
SKEKILYTNYLQGIVSHKFTSNKIIYSYINELTKRCLLSKIQKKEYYENLLALGDLYCQGYIIPW
NNLYPNKFERIHLPGYLFLKEHYWIEKKNINSLKEKNTKYKKIEFSIQILNLSRIILEKKINNSI
NILKNIIWGKLPEIENYKDIKLKINVYKKNNKLVYITNIEKHCCQIGEIDLNNTIIMKNPIDFNE
LQKDLDKLKINCKNVSKIYISKQFLFADILLNKKNNLYNESIFFSKALELIHVFVNYHKIIINND
LLPFSLKYIKIYKKIPLNVILYLICKKVEKNSSYQQYDIIFYDNKGNVCLKLKDLIFKKINKLIN
IDEIFSNL

DipO_Dco

ACP KS10 DH KR ACP KS11 (DH) ACP KR

MIFSDFQDINIKMKKFLENEIINSSKSDLGNDLKNNTKKLVTKNKEKLILNIEKKEKNLLYIYFY
KKIANIVSKILKIPLERMNMQENISRYGVDSIIVTEIIRCISDILNFPIAPTVFFEAKNIEELIN
ILFQRYKEKIKSYFLKNHKRNQVLEEKVNQELKKQKTQENNTEIKDWINKFKFIIASSDSINTSS
DSINTSSDSINYEPIAIISMEGIFPNSPNLQILEKNLRNENDCISEIPLSRWDWKKIFGDPQKGN
FTNVKYGGFAPDIDKFDPIFFGMSPREAELMDPQHRLFIQCVWKLIESAGYAPKSLSGKKIGIFI
GVNLQDYAHIVNKSRKMDSLHLTSLGHMFCPNRLSFYLNIHGPSQVIDTACSSSLVALHRAILSI
QHEGCEMSIAGGANLIISPDMHIMYNKIGMISKDGRCKTFSKYANGYGRSDGIGVVLLKSLRLAE
KDNDNILAVICGSSENHGGMSTSLTAPNPKSQAKLIIEAHNKAKSDPRSIGYIECHGTGTKLGDP
IEVNGLKIAFEELFRINNLSKSNIKFCGLGSIKSNIGHTETSAGIAGVIKTVLSLRNNYLYKSLH
SEDINPIIELDESPFYILQKGCFWKSPILNNHKLPRRAGVSSFGAGGSNAHIVIEEYTKHTEKKF
NFKIIDSDVLIILSAKNIDRLNDIIIEMFYFIKKILEKKKEINLVDIAYTLQVGREAMSERLAII
VNSISQLYLKLEQIYFSIKNKEKLINIISCYYGSIKKEKNIFYVKNENFISDNDINNYFNNKNYK
QLALLWIKGITINWSKLYANKKSKLYRVSLPTYVFAKQSYWIENLNNKRNIDYYLKSHNLSKNSI
SNLGKYYFSAKLTGKEFFLADHIIMKKKILPGVIYLEMVCSAIKEIKIKNISNQFIIQLKNVVWA
QSFFINENCSKILHMKMNQEIDNQISYQIYSEQEKNNINVRKERIIHSQGIAITKNIPIINDINN
INIDNLLENFTKRQLDTNFLLGNICYQMFNDMGINYGPTHQCLEKIYFSQKNKKNPPQVLAKLKL
LKNIKENYKYSKKFMFHPGLVDSGLQACIGFMVYFGYIDFDKKTNKVIINNKNIVSLPFALESFL
FLSQPSTSMWVWIRYSENSLFNSNIQKLDIDFYDKQGKVCICMRGLSSRSQKINNLAKDFTTLVY
KPIWVREDIKIDIKKNKIKNLSNFSIWSHHFIILCDFNNNFPKNIKEDIKKIIPFSKYFYLCIQG
SIQNIYTTISLQIFRKIRKILRSKLQGNILLQIIFIENEKSMFLNGLSGLLQSVSRENTKFYSQL
IGLRDYDTLNLYSSEYIANIIYSNSTGKVYKNKIRYSSIGREVFSWKELNFISYTKAIFSPLKEN
GIYLITGGNGGLGLLITEFIIQKINKGVVVLCGRSFLNDKIRKNINNLSTKNIKIKYYCLDITKK
IEVEEFIKNIIHKYNFINGIIHCAGLLMDNFVYKKSSSEFIKVLEPKVNGTIYLDEATKILNLDF
FILFSSISGSLGSAGQSDYAAANSFLDIFSDYRNKLTSRNQRYGRTISINWPLWSNGGMKMQESI
KNIMLQTSGIVEMPNIHGLDILYKVITLNISRIMVIYGISSRIRNYLENIDKKVINSLSSQENES
SSAINSNNINYNELRNCICKMLSKYISKLMKFSLKDIENDVQLSDYGFDSITFTDLANRLNRKYQ
LELTPTTFFEYPTINALSELLSVQYYDKFIFKFDLKPKFIEKFVKLDKELYLSTDSIEKKEKLLP
INDNKLKNKSHSLLDNKIAIIGISGCFPMSQDINEFWKNLLSGADCISEIPLSRWDWRDIYGDPK
KDANKTNIKWGGFIDNIDFFDASFFNISPREAELMDPQHRLLMQYVWKAIEDAGYSPGSLSGSKT
ALFIGTASSGYGQLIANSNFTIDGYSATGVIDSIGPNRMSYFLNLHGPSEPVETACSSSLVAIHR
ALSAILMEDCEQAIVGGINLIISPETQISFSKAGMLCEDGRCKTFSNKANGYVRGEGVGMLFLKK
LSVAEKDGDHIYGVICGSSENHGGRSSSLTAPNPKAQIEVIKNAYIRANINPQSISYIEAHGTGT
ELGDPIEVNALKSAFRDLNKINNNSEKFIKNAYCGIGSVKTNIGHLELAAGIAGVIKVLLQMKYK
TLVKSLHCEKINPYIILKNSPFYLLHNNCEWKRLRDINGKKIPRRAGISSFGFGGVNAHLVIEEY
SIKKEVFKYESSSIALIVFSAKNIKSLKEYAISLIKFITINNEISIENKIYDHDNICKFLSNIVH
KYISDILNINKKEINFDDQLSQIGLDCIYGIMLLEKLNKKFNIDISFNILLNDHSIISFVNAILL
NYPNLYAIINKKYKKKKILNFDNHFKKIRNDINLFELAYTLQVGRDPMDHRLALTAISFDELLIK
LQAFIDDRINDFQGLYLGRIKENKRIFSIFINDKEIQEALEKWMKCGKLTKLLEIWVLGMKISWE
KLYGESCLYPKKPKRISIPGYPFSKKSYWIKKIFKKQKIYDFDSRISEKLKKLHPLVHENISTLK
YCKFRTFLTGEEFFLSDYSINERKILPNIVYLEMVRAAAVIAQDNFYKSSDVIKFHNIIWSDPFI
FNEEKYIEIELNKNIGNTIQFKIISYPTLNNLDSSKLQIHSQGVISYINSNKYNLPYVDINLILN
KLNIQKFSSVQCYNLFKKFKLIYGPIYRNVYELYVSKKQVLAKLILPELMLVTLKKYILHPSIIG
SALQCIIGLYLDKLNLIFTEENDKELFLSFVIEDIYIFNICIKLMWVHIEYVSNHLTKERIKKLN
INLCNERGVIHIQIKGYILSRFNFEDKEYKINFNNKKINSKNINNSIELILDKSDKNIDKLLYNK

TIEYFKEIFSSLLKYPIDELDINENINSYGINSMAIMELNVILEKQFGTLSKTLFFEFNTLNSLT
NYFISSYHSKLIKLFSINNKKKNKKEKSLSLCQSKIIPKKNDTKIKISSLVKNYSILITTSKWKE
SLISSLPLNKNNKNNIVNKKNYDTHVIFFTNEIILIPGVVCLSLFSIIKKIDYYFFDCAIKIFEY
IQQNLINKNGRILLQIIIQNFQSKICSNIIFPVKFLHSLSGILKTAHIENLNFFGQIIEIKNQNN
KDNFIQEIIQKNALIPFDQNIRYLNGKRYVLYWDKISSTKNIFPFFWKDDGIYLITGGMGKLGLI
FAKEIINKTNNVTLILIGRSKLDSLKQEKLNSLKIRGAEIKYYSIDVTDQTAILELIKSINLKKK
HGLNGILHCAGIIKNNLIYKKSIIEFEEVLLVKVLGAINLDIASRGINLDFMVLFSSISGVFGNY
KCCDYSTANFLINMYSKYRNQLSLSSFIASQVNPKGRILSINFPLCNNDKINIDNYNKELVYKDI
EIKKLNTQNILSIFYYAMHTSYNQIVILQGNLPHLRKLLFLNKKTPQKKSSKKLSDKKTPQKKSS
KKLSDKKTPQKKSSKKLSDKKTPQRKSSKKLSDKKTPQKKSSKKLSDKKTPQKKSSKKLSDKKL
